# STING activation promotes autologous type I interferon–dependent development of type 1 regulatory T cells during malaria

**DOI:** 10.1172/JCI169417

**Published:** 2023-10-02

**Authors:** Yulin Wang, Fabian De Labastida Rivera, Chelsea L. Edwards, Teija C.M. Frame, Jessica A. Engel, Luzia Bukali, Jinrui Na, Susanna S. Ng, Dillon Corvino, Marcela Montes de Oca, Patrick T. Bunn, Megan S.F. Soon, Dean Andrew, Jessica R. Loughland, Jia Zhang, Fiona H. Amante, Bridget E. Barber, James S. McCarthy, J. Alejandro Lopez, Michelle J. Boyle, Christian R. Engwerda

**Affiliations:** 1 QIMR Berghofer Medical Research Institute, Brisbane, Australia.; 2 Griffith University, School of Environment and Science, Nathan, Australia.; 3 University of Queensland, School of Medicine, Brisbane, Australia.; 4 Institute of Experimental Oncology, University Hospital Bonn, Bonn, Germany.; 5 York Biomedical Research Institute, Hull York Medical School, University of York, York, United Kingdom.; 6 Victorian Infectious Diseases Services, Doherty Institute, University of Melbourne, Melbourne, Australia.; 7 Life Sciences Division, Burnet Institute, Melbourne, Australia.

**Keywords:** Infectious disease, Cellular immune response

## Abstract

The development of highly effective malaria vaccines and improvement of drug-treatment protocols to boost antiparasitic immunity are critical for malaria elimination. However, the rapid establishment of parasite-specific immune regulatory networks following exposure to malaria parasites hampers these efforts. Here, we identified stimulator of interferon genes (STING) as a critical mediator of type I interferon production by CD4^+^ T cells during blood-stage *Plasmodium falciparum* infection. The activation of STING in CD4^+^ T cells by cyclic guanosine monophosphate-adenosine monophosphate (cGAMP) stimulated *IFNB* gene transcription, which promoted development of IL-10– and IFN-γ–coproducing CD4^+^ T (type I regulatory [Tr1]) cells. The critical role for type I IFN signaling for Tr1 cell development was confirmed in vivo using a preclinical malaria model. CD4^+^ T cell sensitivity to STING phosphorylation was increased in healthy volunteers following *P*. *falciparum* infection, particularly in Tr1 cells. These findings identified STING expressed by CD4^+^ T cells as an important mediator of type I IFN production and Tr1 cell development and activation during malaria.

## Graphical abstract

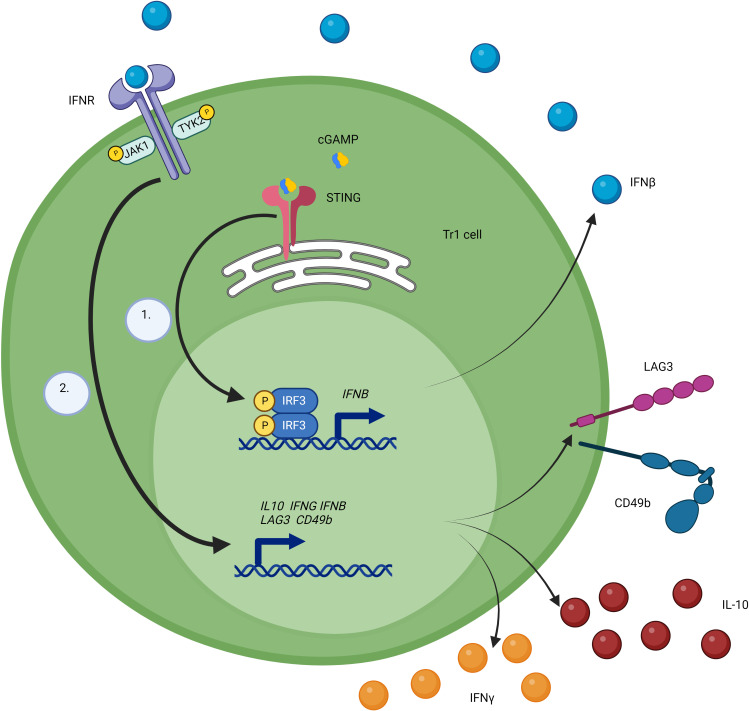

## Introduction

Malaria is a devastating human disease of global importance. It not only caused an estimated 247 million cases and 619,000 deaths in 2021, but also promoted poverty by imposing health care and socioeconomic costs on communities in malaria-endemic areas (1). *Plasmodium falciparum* is responsible for most malaria cases, and despite decades of effort, there is still no licensed vaccine that meets the WHO goal of 75% efficacy against clinical disease (2). Many factors contribute to this failure, including the presence of polymorphic or strain-restricted antigens and suboptimal vaccine formulations and dosing schedules as well as imprinted antiparasitic immune responses that impede rather than enhance vaccine-induced immunity (3–5). In regard to the latter, mounting evidence supports the emergence of potent immune regulatory mechanisms to protect tissues against inflammation following *P*. *falciparum* infection (3, 6, 7).

The production of the antiinflammatory cytokine IL-10 and expression of coinhibitory receptors by parasite-specific CD4^+^ T cells are important components of the immune regulatory networks that arise during malaria (8–10). In fact, IL-10– and IFN-γ–coproducing CD4^+^ T (type I regulatory [Tr1]) cells comprise a substantial fraction of cells responding to parasite antigen stimulation of immune cells from African children living in malaria-endemic areas (11–14). Evidence from both preclinical models (15–17) and controlled human malaria infection (CHMI) studies (3) indicates that type I IFNs are important drivers of Tr1 cell development during malaria.

Type I IFNs play diverse roles in host immune responses during infections and cancer (18, 19). In a preclinical malaria model, type I IFNs suppressed the development of antiparasitic T follicular helper (Tfh) cell responses, thereby limiting parasite-specific antibody production (17). Furthermore, we previously showed that type I IFNs act on dendritic cells to suppress the development of Th1 cells while promoting *Il10* gene transcription in a model of experimental malaria (20). We also demonstrated that in volunteers infected with blood-stage *P*. *falciparum*, type I IFNs suppressed antigen-specific IFN-γ production while promoting parasite-specific IL-10 production (3). Although we identified CD4^+^ T cells along with many other immune cell populations as important sources of type I IFNs, we do not know the mechanism of type I IFN induction during malaria. It is also unknown whether CD4^+^ T cell type I IFN production plays any role in the development of antiparasitic immune responses during malaria, and if so, the identity of relevant cellular and molecular pathways that mediate type I IFN-dependent Tr1 cell development. This information is important if we wish to manipulate Tr1 cell development and/or activity to improve antiparasitic immunity in response to vaccine or drug treatments.

Here, we identified increased expression of *TMEM173* (encoding stimulator of interferon genes [STING]) by Tr1 cells, relative to other CD4^+^ T cell subsets, in volunteers infected with blood-stage *P*. *falciparum*. Furthermore, we uncovered a role for STING in CD4^+^ T cell type I IFN production that promoted the development and activation of Tr1 cells using primary human cells. These results were verified in vivo using a preclinical malaria model in mice. Together, our findings identify a critical cell-signaling axis in CD4^+^ T cells that drives the development and activation of Tr1 cells during malaria, thus providing a potential means for manipulating this key CD4^+^ T cell subset to improve antiparasitic immunity in the context of either vaccination or drug treatment.

## Results

### STING expression by Tr1 cells from volunteers infected with blood-stage P. falciparum.

**Figure 1:**
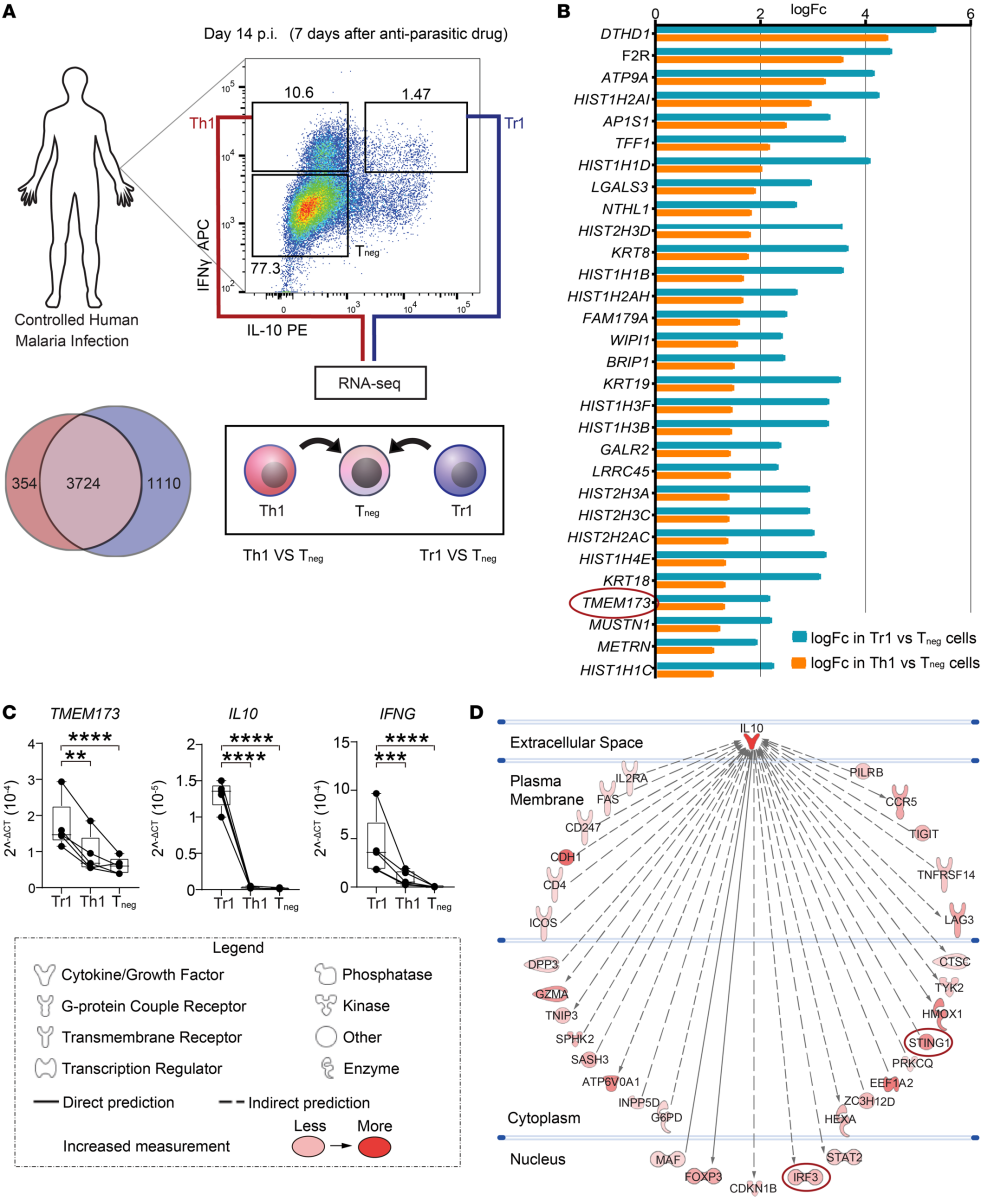
Higher *TMEM173* expression by Tr1 cells compared with Th1 cells during malaria. (**A**) Schematic showing the experimental design for the RNA-Seq analysis of Tr1 and Th1 cells from volunteers participating in CHMI studies with *P*. *falciparum*. (**B**) List of the top 30 differentially upregulated genes between Tr1 and Tneg cells as well as Th1 and Tneg cells from the CHMI study. (**C**) Validation of higher *TMEM173* mRNA expression by Tr1 cells compared with Th1 cells. Human CD4^+^ T cells were isolated from 5 healthy volunteers and then cultured with αCD3ε and αCD28 mAbs plus IL-2 for 3 days. Tr1 and Th1 cells were sorted based on IL-10 and IFN-γ expression, as shown in Figure 1A. *TMEM173* mRNA was detected by qPCR and normalized to the housekeeping gene 18S rRNA. Data were log2-transformed for statistical analysis. Lines connect paired samples, and box shows extent of lower and upper quartiles plus median, while whiskers indicate minimum and maximum data points. *n* = 5 samples. Repeated measures 1-way ANOVA with Šídák’s multiple-comparisons test. **P < 0.01; ***P < 0.001; ****P < 0.0001. (**D**) IPA prediction of genes directly or indirectly associated with IL-10 as well as the extent of the predicted interaction, as indicated by pink to red coloring.

We recently described a transcriptional signature for human Tr1 cells, defined by IL-10 and IFN-γ coproduction, during CHMI studies that distinguished them from IFN-γ–producing CD4^+^ T (Th1) cells (21). Further interrogation of this data set (Figure 1A) revealed that *TMEM173* (ENSG00000184584; encoding STING) was upregulated by Tr1 cells, compared with Th1 cells and other CD4^+^ T cells (Figure 1B). Pattern-recognition receptors are emerging as important mediators of costimulatory pathways in T cells, and STING was recently shown to drive T cell type I IFN production in these cells (22, 23). Thus, given the key immune regulatory roles for type I IFNs previously reported in malaria and, in particular, their role in promoting the transition of Th1 cells to Tr1 cells (3), we examined the role of CD4^+^ T cell STING on the development and activation of Tr1 cells. We first confirmed increased *TMEM173* expression in Tr1 cells in validation experiments (Figure 1C). Next, we employed pathway analysis of the Tr1 cell transcriptomic data to identify molecules that were predicted to interact with IL-10 either directly (direct physical contact) or indirectly (no physical contact, but causes a change in expression). We observed that IL-10 was predicted to indirectly interact with STING as well as interferon regulatory factor 3 (IRF3), a transcription factor downstream of STING activation and a key driver of *IFNB1* gene transcription (24) (Figure 1D). Thus, STING was more highly expressed in Tr1 cells compared with other CD4^+^ T cell subsets and predicted to be associated with IL-10 and type I IFN production by these cells.

### Modulation of CD4^+^ T cell STING activation with CRISPR/Cas9 gene editing.

**Figure 2:**
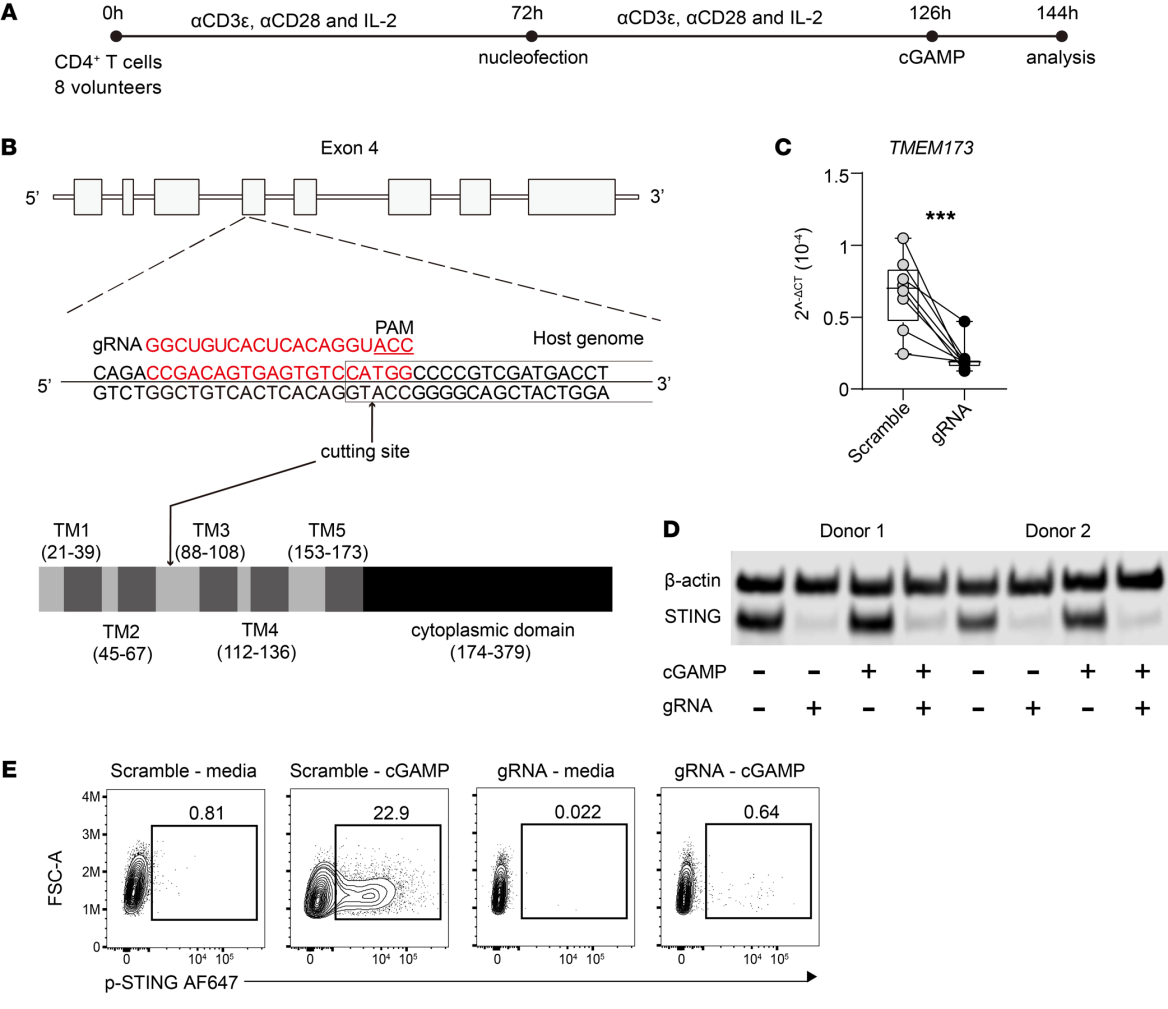
Modulation of CD4^+^ T cell STING expression by CRISPR/Cas9 gene editing. (**A**) CD4^+^ T cells from 8 healthy volunteers were stimulated with αCD3ε and αCD28 mAbs plus IL-2 for 72 hours before nucleofection and then stimulated for another 72 hours under the same conditions. Cells were treated with or without cGAMP for 18 hours before analysis. (**B**) A diagram showing the gene structure of human *TMEM173* and the CRISPR gRNA–targeting sites within exon 4. Domain structure of the human STING protein showing the 4 transmembrane domains of the N-terminal, responsible for ligand binding and protein dimerization. The C-terminal contains the cyclic dinucleotide domain and binding sites for TBK1 and IRF3. (**C**) qPCR validation of *TMEM173* mRNA expression in control and CRISPR gRNA–treated samples. *TMEM173* mRNA was normalized to *18S* rRNA in each sample. Data were log2-transformed for statistical analysis. Lines connect paired samples, and box shows the extent of lower and upper quartiles plus median, while whiskers indicate minimum and maximum data points. *n* = 8 samples. Two-tailed paired *t* test. ***P < 0.001. (**D**) Representative Western blot showing the effect of CRIPSR gRNA modification of *TMEM173* in response to cGAMP stimulation, as indicated. β-Actin was used as a protein-loading control, relative to STING protein levels. (**E**) Representative FACS plots showing loss of STING phosphorylation in control and CRISPR gRNA samples treated, as indicated.

To investigate the role of STING in human CD4^+^ T cells, we employed CRISPR/Cas9 gene editing of *TMEM173*. We used a previously reported protocol (25, 26) to optimize editing STING expression in primary CD4^+^ T cells isolated from peripheral blood of healthy volunteers (Supplemental Figure 1, A and B; supplemental material available online with this article; https://doi.org/10.1172/JCI169417DS1). CD4^+^ T cells were cultured for 3 days with anti-CD3ε and anti-CD28 mAbs in the presence of recombinant IL-2 (Figure 2A) before being edited with a guide RNA (gRNA) targeting the *TMEM173* gene (Figure 2B). Following a further 3 days of cell culture under the same conditions, approximately 73% of cells had excisions in exon 4 of the *TMEM173* gene (Supplemental Figure 1, C and D) and reduced *TMEM173* mRNA accumulation (Figure 2C) and STING protein levels (Figure 2D). We confirmed the loss of STING by showing stimulation with the STING agonist cyclic guanosine monophosphate-adenosine monophosphate (cGAMP), resulting in negligible detection of phosphorylated STING (p-STING) (Figure 2E). Thus, we were able to modify *TMEM173* using CRISPR/Cas9 gene editing, resulting in CD4^+^ T cells that were unable to respond to stimulation with cGAMP.

### CD4^+^ T cell STING is required for Tr1 cell development.

**Figure 3:**
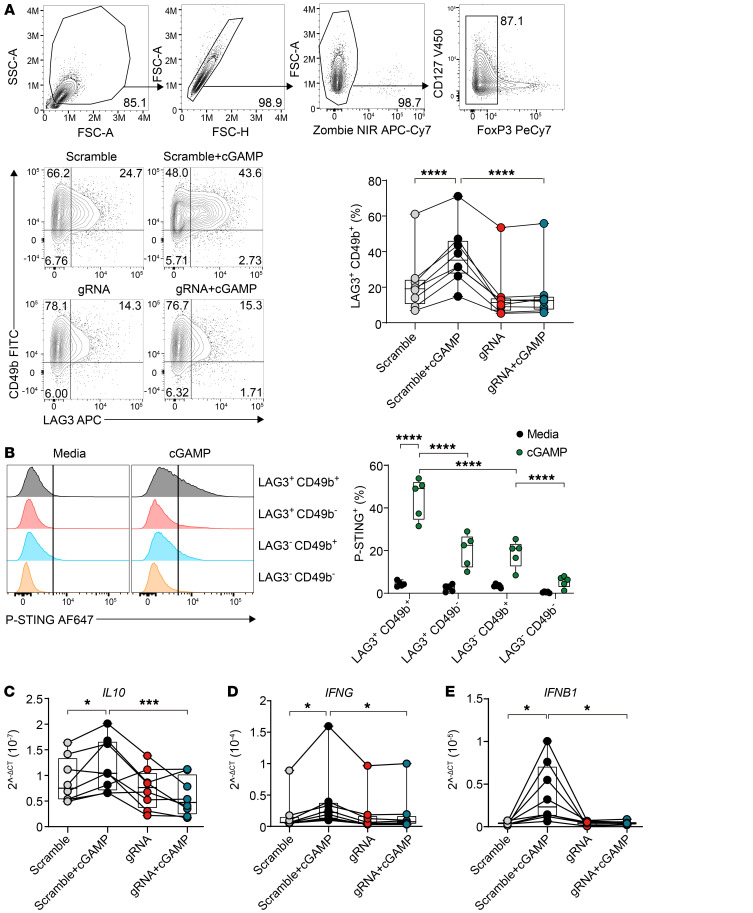
CD4^+^ T cell STING activation promotes Tr1 cell development. Human CD4^+^ T cells were cultured and subjected to CRISPR/Cas9 *TMEM173* gene editing as shown in Figure 2. (**A**) Gating strategy used to assess changes in human CD4^+^ T cells. Cells were gated on single cells, live cells, and conventional CD4^+^ T cells (FoxP3^–^) before further analysis. Representative plots and enumeration showing the frequency of LAG3^+^CD49b^+^ CD4^+^ T cells following CRISPR/Cas9-mediated modification of *TMEM173* expression. Lines connect paired samples, and box shows extent of lower and upper quartiles plus median, while whiskers indicate minimum and maximum data points. (**B**) Representative histograms and enumeration showing the frequencies of p-STING–positive LAG3^+^CD49b^+^, LAG3^+^CD49b^–^, LAG3^–^CD49b^+^, and LAG3^–^CD49b^–^ CD4^+^ T cell subsets. Box shows the extent of lower and upper quartiles plus median, while whiskers indicate minimum and maximum data points. (**C**–**E**) Expression of *IL10*, *IFNG*, and *IFNB1* in the control and *TMEM173*-modified cells with and without cGAMP activation was measured by qPCR. Lines connect paired samples, and box shows the extent of lower and upper quartiles plus median, while whiskers indicate minimum and maximum data points. *n* = 8 (**A**, **C**–**E**); *n* = 5 (**B**). Repeated measures 2-way ANOVA with Šídák’s multiple-comparisons test. *P < 0.05; ***P < 0.001; ****P < 0.0001.

To identify Tr1 cells without the need for stimulation with strong mitogens such as phorbol myristate acetate (PMA) to detect IL-10 and IFN-γ, we used LAG3 and CD49b, which have previously been shown to be highly expressed by Tr1 cells (27, 28). Tr1 cells identified by LAG3 and CD49b coexpression peaked at day 4 after stimulation of CD4^+^ T cells with anti-CD3ε and anti-CD28 mAbs plus IL-2, and consistent with previous studies (27, 29), LAG3 and CD49b coexpressing cells produced the highest amounts of IL-10 and IFN-γ as well as their transcripts, although IL-10 production peaked 24 hours after stimulation (Supplemental Figure 2). Thus, detection of LAG3 and CD49b coexpression on CD4^+^ T cells in culture was more reliable than IL-10 production over time (Supplemental Figure 2). However, this latter observation indicates that some caution must be used when assessing Tr1 cells with the former cell-surface markers. Nevertheless, the development of LAG3^+^CD49b^+^ Tr1 cells was enhanced by cGAMP (Figure 3A), while there was a decrease in the frequency of other CD4^+^ T cell subsets, although additional markers for chemokine receptors and transcription factors will be needed to identify these subsets (Supplemental Figure 3, A–C). Regardless, these results show that cGAMP activation of STING promoted Tr1 cell development and that CRISPR/Cas9 gene editing of *TMEM173* in human CD4^+^ T cells limited this process (Figure 3A). To determine whether Tr1 cells were the main CD4^+^ T cell subset responding to cGAMP, we examined STING phosphorylation following cGAMP stimulation of activated CD4^+^ T cells. Indeed, we observed the highest frequency of p-STING^+^ cells among LAG3^+^CD49b^+^CD4^+^ T cells (Figure 3B). Stimulation of CD4^+^ T cells with cGAMP also increased STING-dependent transcription of *IL10*, *IFNG*, and *IFNB1*, but following *TMEM173* gene editing, this was abrogated (Figure 3, C–E). Notably, cGAMP stimulation resulted in no marked increase to the transcription of other type I IFN family members (Supplemental Figure 3D), suggesting selective induction of *IFNB1* among the type I IFN family of genes in CD4^+^ T cells following STING activation. We attempted to measure IL-10 protein in cell-culture supernatants, but levels were around or below levels of detection. Nevertheless, these results show that CD4^+^ T cell STING promotes Tr1 cell development and that its activation in these cells drives *IL10*, *IFNG*, and *IFNB1* transcription.

### STING-dependent IFN-β1 production by CD4^+^ T cells drives Tr1 cell development.

**Figure 4:**
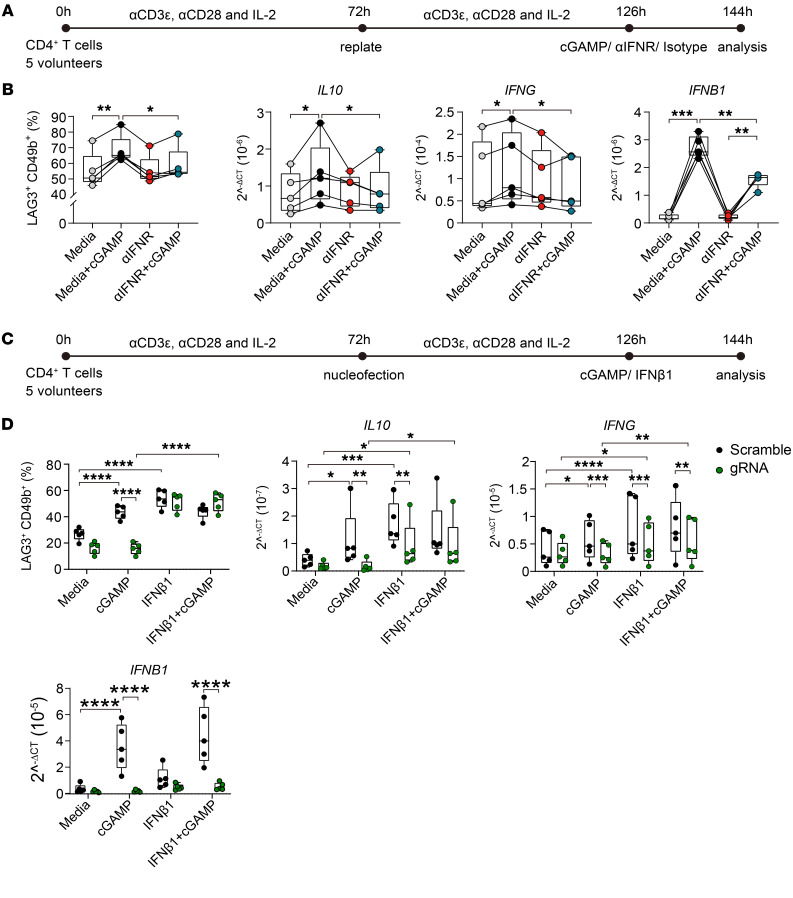
STING-dependent IFN-β1 production by CD4^+^ T cells drives Tr1 cell development. (**A**) CD4^+^ T cells were stimulated with αCD3ε and αCD28 mAbs plus IL-2, as shown, prior to treating with an antibody against type I IFNR (αIFNR), isotype control mAb, or cGAMP for 18 hours before analysis, as indicated. (**B**) Cells were gated on conventional CD4^+^ T cells (FoxP3^–^), as shown in Figure 3A. The frequency of LAG3^+^CD49b^+^ CD4^+^ T cells as well as *IL10*, *IFNG*, and *IFNB1* mRNA levels in each treatment group was measured. qPCR data were normalized to the housekeeping gene *18S* rRNA. (**C**) CRISPR/Cas9 modification of *TMEM173* in CD4^+^ T cells stimulated with αCD3ε and αCD28 mAbs plus IL-2, as shown, prior to treating with 100 ng/μl recombinant IFN-β1 and/or 30 μg/ml cGAMP 18 hours before analysis, as indicated. (**D**) Frequency of LAG3^+^CD49b^+^ CD4^+^ T cells and *IL10*, *IFNG*, and *IFNB1* mRNA were measured in CD4^+^ T cells treated as indicated. (**B** and **D**) Lines connect paired samples, and box shows extent of lower and upper quartiles plus median, while whiskers indicate minimum and maximum data points. *n* = 5. Repeated measures 2-way ANOVA with Šídák’s multiple-comparisons test. *P < 0.05; **P < 0.01; ***P < 0.001; ****P < 0.0001.

Type I IFNs not only have potent antiviral activities, but also modulate CD4^+^ T cell responses during experimental and clinical malaria, including suppressing Th1 and Tfh cell responses (3, 15, 17, 20). Given the strong STING-dependent induction of *IFNB1* by Tr1 cells, we next examined the requirement of type I IFN production for Tr1 cell activation or development (Figure 4A). Following CD4^+^ T cell activation, as above, we found that STING-dependent expansion of Tr1 cells was abrogated by blocking type I IFN signaling with an antibody directed against the type I IFN receptor (IFNR) (Figure 4B). We also observed reduced *IL10* and *IFNG* transcription when type I IFN signaling was blocked (Figure 4B) as well as diminished *IFNB1* induction following IFNR blockade (Figure 4B). To directly link CD4^+^ T cell–autologous STING-dependent IFN-β1 production with Tr1 cell activation and development, we tested to determine whether Tr1 cell development from STING-deficient CD4^+^ T cells could be rescued by exogenous IFN-β1 (Figure 4C), and indeed this was the case (Figure 4D). We also found that supplementation of CD4^+^ T cells with IFN-β1 alone induced *IL10* and *IFNG* transcription (Figure 4D). Together, these results show that STING-dependent IFN-β1 production by CD4^+^ T cells promotes Tr1 cell development and activation.

### CD4^+^ T cell STING is required for optimal IFN-γ and IL-10 production in experimental malaria.

**Figure 5:**
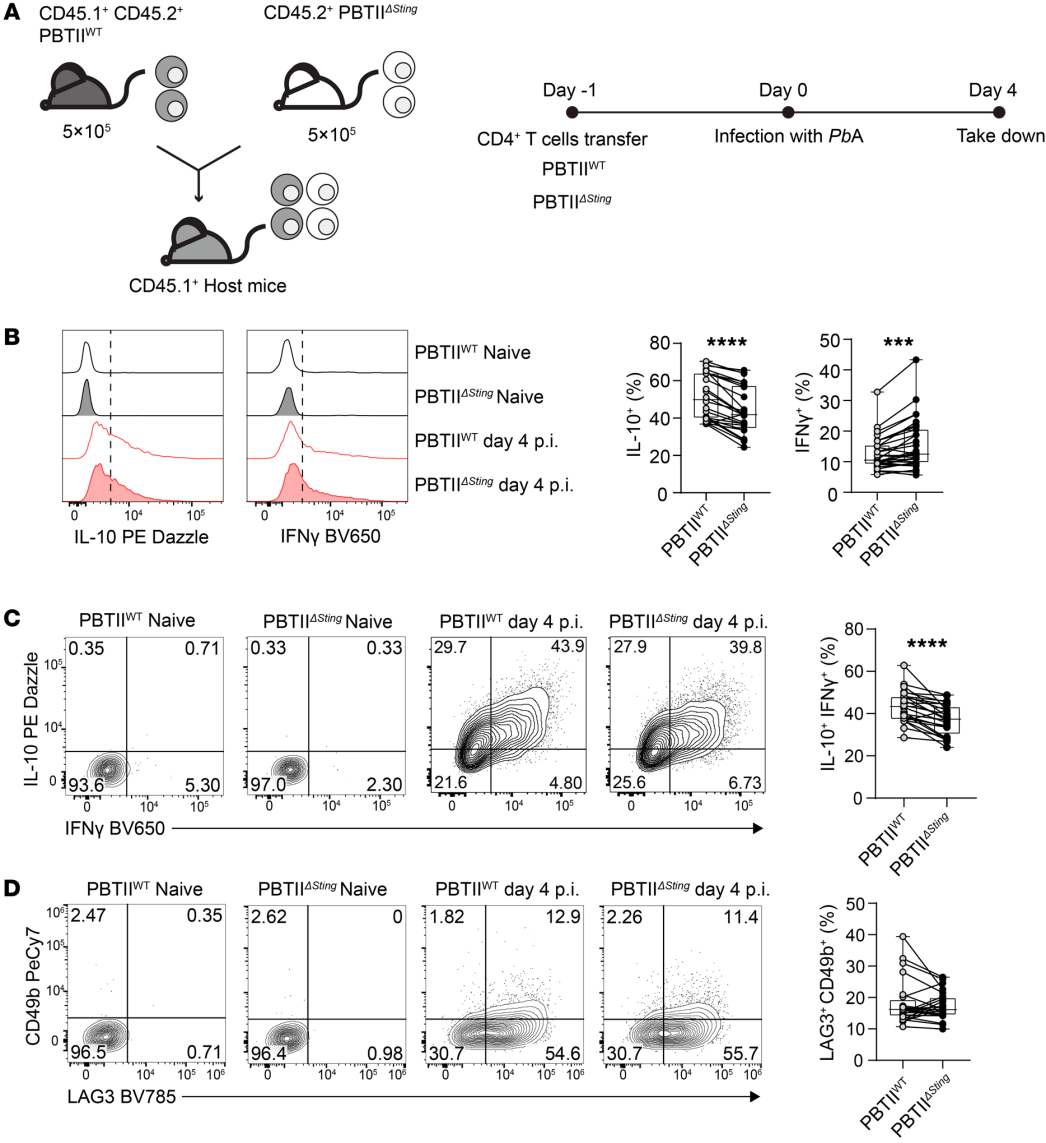
CD4^+^ T cell STING is required for Tr1 cell development in experimental malaria. (**A**) 5 × 10^5^ CD45.2^+^ PbTII^ΔSting^ and 5 × 10^5^ CD45.1^+^ CD45.2^+^ PbTII^WT^ cells were transferred into the *Ptprca* (CD45.1^+^) recipient mice at day –1. The mice were infected with *P*. *berghei* ANKA (*Pb*A) on day 0 and were assessed on day 4. (**B**) Representative histograms and enumeration showing the IL-10– and IFN-γ–producing PbTII^WT^ and PbTII^ΔSting^ cells. (**C** and **D**) Representative plots and enumeration showing the frequencies of IL-10^+^IFN-γ^+^ and LAG3^+^CD49b^+^ CD4^+^ T cells, respectively. Data in each plot were pooled from 3 independent experiments. Lines connect paired samples, and box shows extent of lower and upper quartiles plus median, while whiskers indicate minimum and maximum data points. *n* = 24. Two-tailed paired *t* test. ***P < 0.001; ****P < 0.0001.

To extend the above studies to an in vivo setting, we used an experimental model of severe malaria caused by infection of C57BL/6 mice with *P*. *berghei* ANKA (*Pb*A). We employed PbTII mice (30, 31), a TCR transgenic mouse line that produces CD4^+^ T cells specific for I-A^b^–restricted *PbA* heat shock protein 90 expressed by all rodent and human *Plasmodium* species, and crossed these with *Tmem173*-deficient mice (32, 33) to generate STING-deficient PbTII cells (PbTII^ΔSting^). WT control PbTII cells were generated by crossing PbTII TCR transgenic mice with congenic (CD45.1) C57BL/6 mice to produce mice expressing both *cd45.1* and *cd45.2* alleles (PbTII^WT^). We then isolated PbTII^ΔSting^ and PbTII^WT^ cells from these animals to test the need for CD4^+^ T cell STING for Tr1 cell development in vivo. We transferred these cells at an equal mix (10^6^ total) into congenic (CD45.1) C57BL/6 recipient mice the day before *Pb*A infection (Figure 5A). We then measured cell frequencies and cytokine production at day 4 post infection (p.i.) in the spleen, when Th1 cell responses peak in this tissue in this model (Supplemental Figure 4), and found a decrease in the proportion of splenic PbTII^ΔSting^ cells producing IL-10 and an increase in those producing IFN-γ relative to control PbTII^WT^ cells (Figure 5B and Supplemental Figure 5, A and B). Furthermore, there was a decrease in the proportion of IL-10^+^IFN-γ^+^PbTII^ΔSting^ Tr1 cells, compared with control PbTII^WT^ cells (Figure 5C). A decreased frequency of PbTII^ΔSting^ cells producing granzyme B (GzmB) and perforin relative to PbTII^WT^ cells was also observed (Supplemental Figure 6, A–C). The expression of these cytotoxic molecules has previously been associated with both mouse and human Tr1 cells (34, 35). However, differences in Tr1 cells defined by LAG3 and CD49b expression were less consistent between PbTII^ΔSting^ and PbTII^WT^ cells (Figure 5D). It should be noted that the frequency of Tr1 cells defined by LAG3 and CD49b coexpression was lower than when these cells were identified by IFN-γ and IL-10 coexpression (Figure 5, B and C), indicating that the former marker set may not capture all Tr1 cells. Regardless, this result suggests that alternative type I IFN cellular sources (not adoptively transferred PbTII^ΔSting^ cells) were driving expression of LAG3 and CD49b, but not the changes in cytokine, GzmB, or perforin production by CD4^+^ T cells in vivo. Hence, these results indicate that CD4^+^ T cell STING promotes IL-10 production while suppressing IFN-γ production in a cell-intrinsic manner in vivo in experimental malaria.

### Type I IFN signaling in CD4^+^ T cells drives Tr1 cell development in experimental malaria.

**Figure 6:**
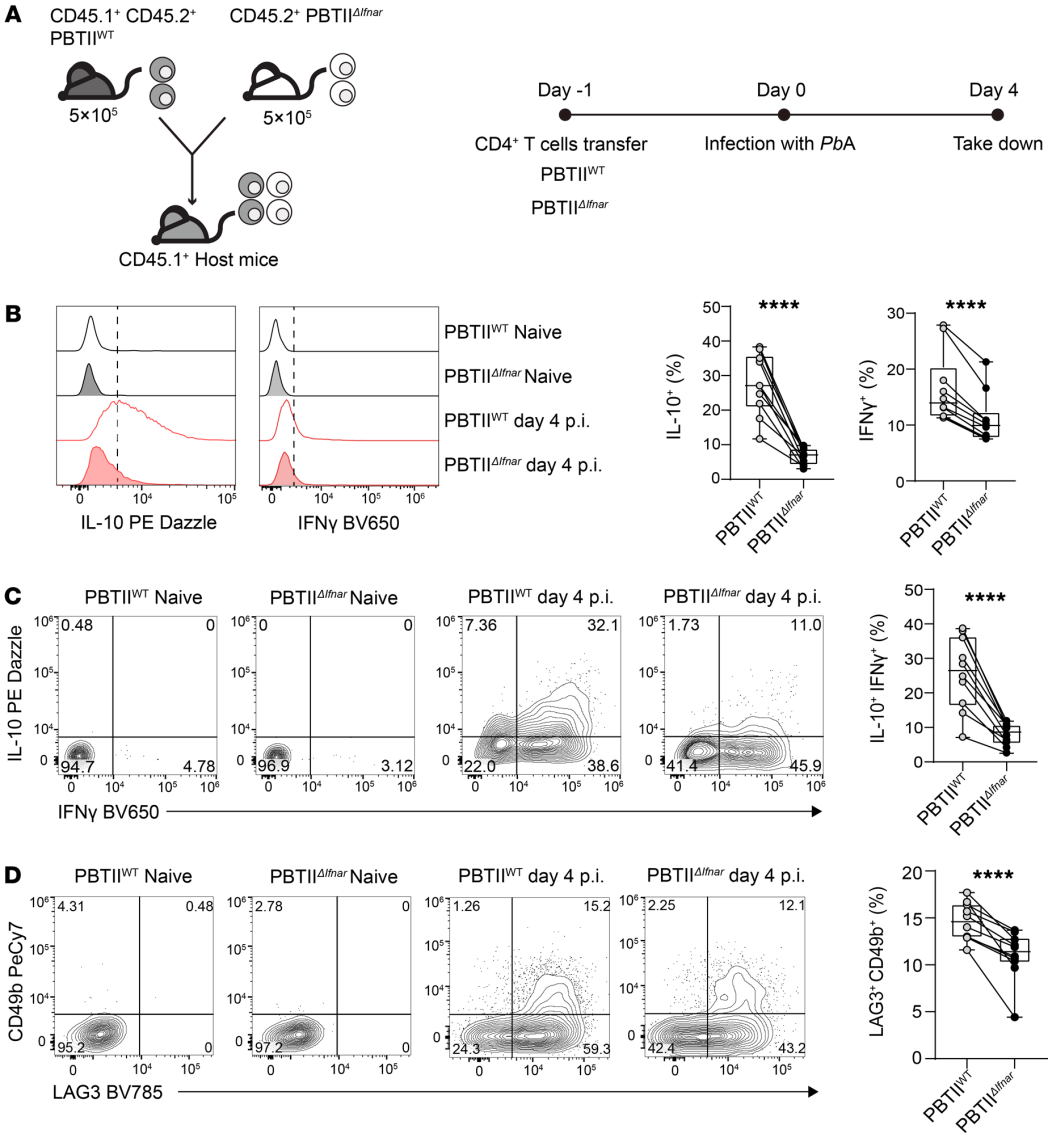
Type I IFN signaling to CD4^+^ T cells drives Tr1 cell development in experimental malaria. (**A**) 5 × 10^5^ CD45.2^+^ PbTII^ΔIfnar^ and 5×10^5^ CD45.1^+^ CD45.2^+^ PbTII^WT^ cells were transferred into the *Ptprca* (CD45.1^+^) recipient mice at day –1. The mice were infected with *P*. *berghei* ANKA (*Pb*A) on day 0 and were assessed on day 4. (**B**) Representative histograms and enumeration showing IL-10– and IFN-γ–producing PbTII^WT^ and PbTII^ΔIfnar^ cells. (**C** and **D**) Representative plots and enumeration showing the frequencies of IL-10^+^IFN-γ^+^ and LAG3^+^CD49b^+^ CD4^+^ T cells, respectively. Data are pooled from 2 independent experiments. Lines connect paired samples, and box shows extent of lower and upper quartiles plus median, while whiskers indicate minimum and maximum data points. *n* = 10. Two-tailed paired *t* test. ****P < 0.0001.

We showed that STING-dependent IFN-β1 production by CD4^+^ T cells drives human Tr1 cell development and activation in vitro (Figure 4). To determine whether cell-intrinsic type I IFN signaling was required for Tr1 cell development and activation in vivo, we again employed the above model of experimental malaria. However, instead of using PbTII^ΔSting^ cells, we crossed PbTII mice with *Ifnar*-deficient mice (36, 37) to generate *Ifnar*-deficient PbTII cells (PbTII^ΔIfnar^) that lacked the ability to receive stimulation by type I IFNs. Following transfer of an equal mix (10^6^ total) of PbTII^ΔIfnar^ and PbTII^WT^ cells into congenic C57BL/6 recipient mice the day before *Pb*A infection, cell frequencies and cytokine production were measured in the spleen at day 4 p.i. (Figure 6A). We found a decreased proportion of splenic PbTII^ΔIfnar^ cells producing IL-10 or IFN-γ and a decrease in cells producing IFN-γ plus IL-10 as well as LAG3^+^CD49b^+^ Tr1 cells relative to control PbTII^WT^ cells (Figure 6, B–D). As previously observed, the decrease in PbTII^ΔIfnar^ Tr1 cell frequency was also associated with a decreased frequency of Tr1 cells producing GzmB and perforin, relative to PbTII^WT^ Tr1 cells (Supplemental Figure 7). Hence, these results show that CD4^+^ T cell–intrinsic type I IFN signaling is required for optimal IL-10 and IFN-γ production as well as Tr1 cell development in vivo in experimental malaria. Furthermore, the results suggest that type I IFN signaling plays distinct roles in CD4^+^ T cell IFN-γ production, whereby it is needed for induction of IFN-γ production, but also promotes the STING/IL-10 axis, which in turn suppresses IFN-γ production by Th1 cells.

### Tr1 cells from humans infected with P. falciparum are more sensitive to STING activation.

**Figure 7:**
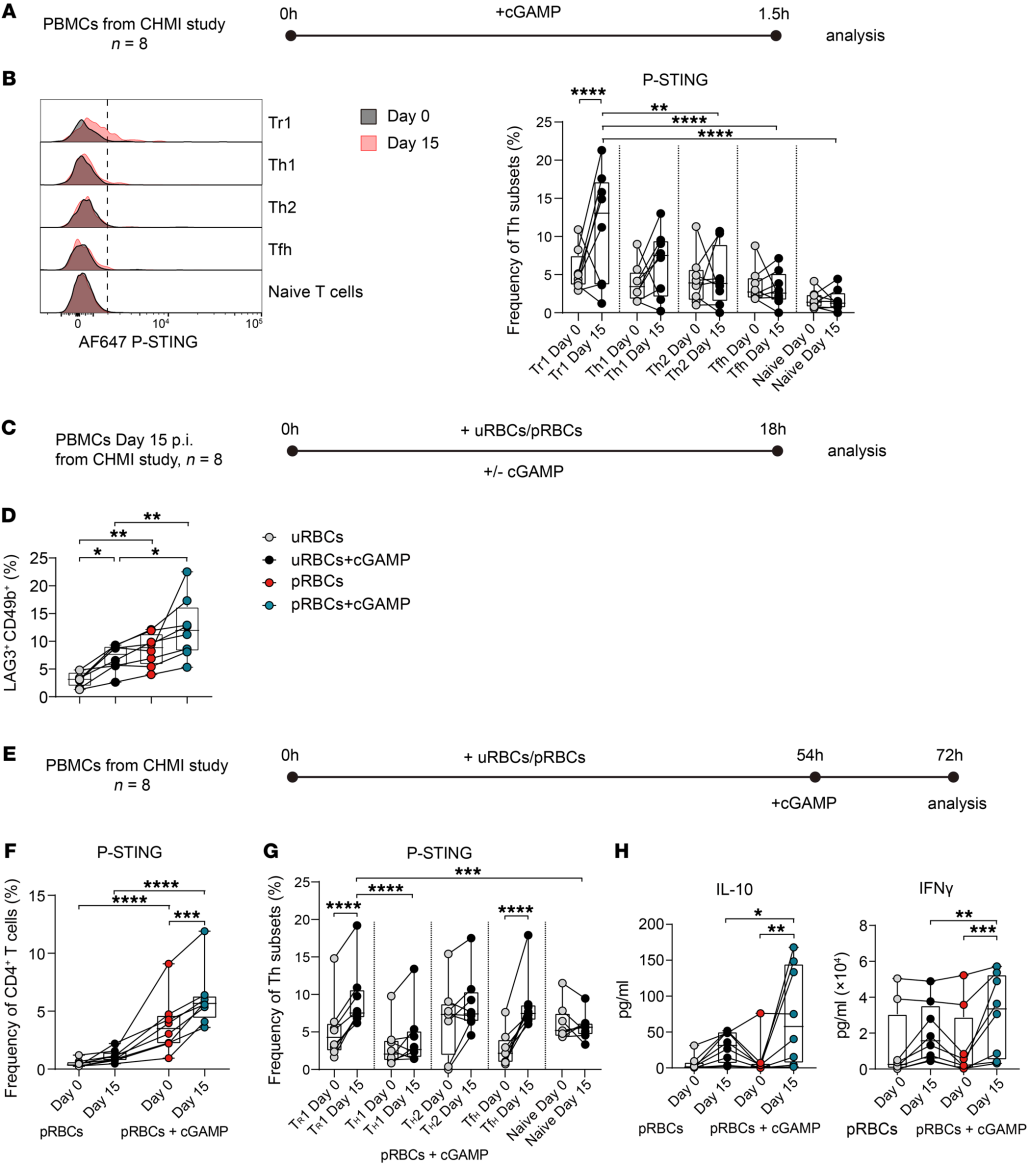
Tr1 cells from humans infected with *P*. *falciparum* are more sensitive to STING activation. (**A**) PBMCs were isolated from volunteers participating in a CHMI study with *P*. *falciparum* at day 0 and 15 p.i. and stimulated with or without cGAMP for 1.5 hours before analysis. (**B**) Representative histogram and enumeration showing the expression of p-STING in different Th cell subsets on days 0 and 15 p.i. (**C**) PBMCs were stimulated with uRBCs or pRBCs for 18 hours with or without cGAMP before analysis of CD4^+^ T cell subset frequencies. (**D**) Tr1 (LAG3^+^CD49b^+^) cell frequencies as a percentage of CD4^+^ T cells are shown. (**E**) PBMCs were stimulated with uRBCs or pRBCs for 72 hours and stimulated with cGAMP 18 hours before analysis. (**F**) Frequency of p-STING^+^CD4^+^ T cells in the presence of pRBCs with or without cGAMP at days 0 and 15 p.i. (**G**) Expression of p-STING in different CD4^+^ T cell subsets in the presence of pRBCs and cGAMP at days 0 and 15 p.i. (**H**) IL-10 and IFN-γ produced in the cell-culture supernatant in the presence of pRBCs with or without cGAMP at days 0 and 15 p.i. Lines connect paired samples, and box shows extent of lower and upper quartiles plus median, while whiskers indicate minimum and maximum data points. (**B**, **D**, **F**, **G** and **H**) *n* = 8. Repeated measures 2-way ANOVA with Šídák’s multiple-comparisons test. *P < 0.05; **P < 0.01; ***P < 0.001; ****P < 0.0001.

Our results above indicate that STING-dependent IFN-β1 production by human CD4^+^ T cells drives Tr1 cell development in vitro and a similar STING-dependent pathway promotes Tr1 cell development in vivo in experimental malaria. Therefore, we hypothesized that CD4^+^ T cell STING would be more readily activated in parasite-specific CD4^+^ T cells from humans infected with *P*. *falciparum*. To test this, we examined peripheral blood CD4^+^ T cells from volunteers participating in CHMI studies with *P*. *falciparum* prior to infection and at day 15 p.i. (7 days after start of drug treatment), as we previously showed this was when Tr1 cell responses peaked (3). We first assessed p-STING in CD4^+^ T cell subsets following stimulation of PBMCs with cGAMP for 1.5 hours, as previously described (38), to evaluate ex vivo sensitivity to STING activation (Figure 7A and Supplemental Figure 8A). The frequency of cells containing p-STING was heterogeneous among volunteers, but peaked in Tr1 cells in most volunteers at day 15 p.i. (Supplemental Figure 8B). At this time, the frequency of Tr1 cells expressing p-STING was greater than in other CD4^+^ T cell subsets examined (Figure 7B). We next determined whether parasite-specific CD4^+^ T cells were more sensitive to STING-mediated development into Tr1 cells. PBMCs collected from CHMI volunteers 15 days p.i. were cultured for 18 hours with uninfected red blood cells (uRBCs) and *P*. *falciparum*–parasitized red blood cells (pRBCs) with or without cGAMP (Figure 7C). PBMCs were used, rather than purified CD4^+^ T cells, so that APCs were available for parasite antigen presentation. However, the use of PBMCs meant that the contribution of antigen-presenting cell activation in these assays to subsequent IL-10 production by CD4^+^ T cells could not be discounted. Nevertheless, day 15 p.i. is when antiparasitic CD4^+^ T cell responses peak in CHMI volunteers (3), and we restricted the cell-culture time to 18 hours to try and capture the ex vivo potential of these cells for Tr1 cell development. cGAMP stimulation increased the frequency of Tr1 cells (LAG3^+^CD49b^+^) at this time point in the presence of uRBCs and pRBCs, but this increase was greatest in the presence of pRBCs, suggesting parasite-specific CD4^+^ T cells were more capable of STING-dependent Tr1 cell development (Figure 7D).

We next examined how different parasite-specific CD4^+^ T cell subsets differed in their sensitivity to STING activation following stimulation by culturing PBMCs from CHMI volunteers taken prior to infection and at day 15 p.i. for 72 hours with uRBCs or pRBCs with and without cGAMP (Figure 7E). The cGAMP was added 18 hours prior to cell assessment because we know STING is activated over this time period (Figure 2), and we wanted to allow sufficient time for STING activation to influence cytokine production. As anticipated from results above, a greater frequency of CD4^+^ T cells responded to cGAMP activation following infection with *P*. *falciparum* (Figure 7F), supporting increased sensitivity of parasite-specific CD4^+^ T cells to STING activation. Furthermore, the frequency of Tr1 cells (LAG3^+^CD49b^+^) responding to parasite antigen and cGAMP activation following *P*. *falciparum* infection was again substantially increased (Figure 7G). However, in this assay involving longer cell exposure to parasite antigen, Tfh cells (CXCR5^+^ PD1^+^) also responded to cGAMP activation following *P*. *falciparum* infection, but not other CD4^+^ T cell subsets examined (Figure 7G). The increase in Tr1 cell STING phosphorylation was associated with a marked increase in antigen-stimulated IL-10 and IFN-γ production following activation with cGAMP (Figure 7H). Thus, Tr1 cells from humans infected with *P*. *falciparum* have increased sensitivity to STING agonists, and following STING activation, this was associated with increased IL-10 production.

## Discussion

In this study, we show STING is expressed by human Tr1 cells following *P*. *falciparum* infection. Furthermore, activation of STING by cGAMP can drive CD4^+^ T cell IFN-β1 production that promotes autologous Tr1 cell expansion and/or maintenance as well as increased IL-10 and IFN-γ production following activation.

Tr1 cells have emerged as an important CD4^+^ T cell subset in numerous clinical contexts (39, 40). In malaria, Tr1 cells develop in healthy volunteers infected with *P*. *falciparum* soon after treatment with antiparasitic drugs (3) and were observed in African children with malaria (11–14). The ability of Tr1 cells to produce IL-10 and express coinhibitory receptors makes them important for protecting tissues from inflammation (39, 40). However, their immunosuppressive functions may also dampen antiparasitic immunity, thereby impeding the development of natural or vaccine- or drug-mediated protection against disease (12, 41, 42). Previous studies have shown that IL-27 drives the balance between Th1 and Tr1 cell development in mice with experimental malaria (43–46) and involves the transcription factors cMaf (47) and Blimp-1 (42). Our findings identify STING expressed by CD4^+^ T cells as another important molecule in Tr1 cell development. Of note, we recently reported that IL-27 had a limited effect on IL-10 production by human CD4^+^ T cells and instead played an important role in promoting coinhibitory receptor expression by specific CD4^+^ T cell subsets (21). Thus, it is likely that, although type I IFNs and IL-27 both affect Tr1 cell development and functions, their roles are distinct.

We identified cGAMP-mediated activation of STING as critical for CD4^+^ T cell type I IFN production. Furthermore, we showed this promoted an autologous regulatory loop that promoted Tr1 cell development and expansion. Of note, we only detected *IFNB* mRNA in human CD4^+^ T cells following activation with cGAMP, and no other members of the type I IFN cytokine family were examined. Recent studies have shown that in mice, cGAMP can be produced and secreted by cells, then taken up by surrounding cells via the volume-regulated anion channel LRRC8C expressed by T cells to activate STING (48, 49). Hence, one possible mechanism for STING activation in CD4^+^ T cells is via phagocytic cells capturing pRBCs and detecting parasite DNA, as previously described (50, 51), then secreting cGAMP that is taken up by CD4^+^ T cells to activate STING and drive Tr1 cell development during malaria.

In addition to their key roles in innate immune cells, pattern-recognition receptors are being increasingly recognized as playing important roles in the activation and fate of T cells (22). Previous studies have shown that STING activation in CD4^+^ T cells can induce apoptosis (52, 53) or suppress proliferation (54). The latter role for STING is mediated via the inhibition of the metabolic checkpoint kinase, mechanistic target of rapamycin (mTOR), while simultaneously stimulating type I IFN production (23). Although we know type I IFNs induce IL-10 production (55, 56), our knowledge about the cellular and molecular mechanisms responsible is incomplete. Our findings support a model whereby CD4^+^ T cell IFN-β production in response to STING activation by cGAMP stimulates IL-10 production as well as Tr1 cell development. However, although we identify an important immune regulatory role for STING-mediated, cell-autologous IFN-β1 production by human CD4^+^ T cells in vitro, our experiments in mice infected with *Pb*A showed that alternate cellular sources of type I IFNs could also affect CD4^+^ T cell activation and effector functions in vivo. Parasite-specific CD4^+^ T cells deficient in STING displayed only a partial defect in Tr1 cell development, based on the 2 definitions used to define these cells (IFN-γ and IL-10 coproduction, LAG3^+^ and CD49b^+^), while this defect was more pronounced in type I IFNR–deficient PbTII cells. These findings and the observation that STING-dependent Tr1 cell development could be rescued by addition of recombinant IFN-β1 to STING-deficient human CD4^+^ T cells, indicates that type I IFNs from different cell sources could drive CD4^+^ T cell activation and differentiation pathways. Most cells are capable of producing type I IFNs (18, 19, 50, 51), and we have previously reported that multiple immune cell populations from volunteers participating in CHMI studies are able to produce these cytokines (3).

There are several potential limitations in our study, including a strict definition for Tr1 cells in malaria. We recently reported that Tr1 cells that emerge following *P*. *falciparum* infection represent a heterogeneous cell population, based on the expression of coinhibitory receptors (21). Furthermore, although we identify CD4^+^ T cells as important cellular sources of type I IFNs during malaria for driving Tr1 cell development, we did not evaluate the contributions of other cellular sources to this process or the relationship of this cytokine signaling pathway to other immune regulatory pathways in malaria. We also didn’t identify CD4^+^ T cell STING agonists active during malaria or their cellular source. Finally, the effects of CD4^+^ T cell STING activation on preexisting Th1 and Tr1 cells from individuals living in malaria-endemic areas were not evaluated.

In summary, we have uncovered a Tr1 cell–development pathway that can be targeted in mice and humans during malaria to alter the balance between parasite-specific Th1 and Tr1 cells. These findings have potential applications in strategies designed to improve vaccine efficacy and/or improve antiparasitic responses following drug treatment, thereby addressing a major bottleneck in efforts to eliminate malaria.

## Methods

### Human primary cells.

PBMCs were isolated using Ficoll-Hypaque (GE Healthcare Bio-Sciences AB), according to the manufacturer’s instructions. PBMCs were suspended in freezing media (90% [v/v] FBS; Gibco/Thermo Fischer Scientific) and 10% (v/v) DMSO (Sigma-Aldrich) and stored at –80°C. PBMCs were thawed and washed with RPMI 1640 media (Life Technologies) and rested in complete media (10% [v/v] FCS, 100 U/ml penicillin, 100 μg/ml streptomycin [penicillin-streptomycin], 1× GlutaMAX, 1× nonessential amino acids, 1 mM sodium pyruvate, 5 mM HEPES [Gibco, Thermo Fisher Scientific] and 0.05 mM 2-mercaptoethanol [Sigma-Aldrich]) in RPMI 1640 containing l-glutamine (Gibco, Thermo Fisher Scientific) for 40 minutes before any further manipulation. Human CD4^+^ T cells were negatively selected from PBMCs using an EasySep Human CD4^+^ T Cell Enrichment Kit, according to the manufacturer’s protocol (STEMCELL Technologies).

### Nucleofection.

Plates (48- or 96-well, flat bottom) were coated with 10 μg/ml αCD3ε mAbs (BioLegend) and incubated at 37°C for 5 hours, then stored at 4°C overnight. Human CD4^+^ T cells were stimulated with plate-bound αCD3ε mAbs and 5 μg/ml soluble αCD28 mAbs (BioLegend) plus 200 U/ml IL-2 (Miltenyi Biotec) for 3 days. 225 μl P3 buffer (Amaxa P3 Primary Cell 96-well Nucleofector Kit, Lonza) and 50 μl of supplement (Lonza) were mixed to make 275 μl nucleofection buffer. CD4^+^ T cells were then washed with PBS and suspended in P3 buffer (Lonza) to achieve a final concentration of 5 × 10^5^ to 1 × 10^6^ cells per 20 μl. 2.4 μl CRISPR gRNA (gRNA, Lonza; Supplemental Table 1) (100 μM) and 2 μl Cas9-NLS (80 μM) (a gift from Chris Jeans, QB3 MacroLab, University of California, Berkeley, California, USA) were mixed and incubated for 40 minutes at 37°C. After incubation, 4.4 μl gRNA-Cas9 complex was added to a 20 μl cell suspension in P3 buffer, transferred into a Nucleovette (Lonza), and placed on an Amaxa Nucleofector and 96-well Shuttle (Lonza) with the program EH115. After electroporation, cells were rested in warm media for 20 minutes at 37°C and stimulated with 25 μl/ml Immunocult (STEMCELL Technologies) and 200 U/ml IL-2 for another 3 days.

### Mice.

C57BL/6/J (WT) mice were purchased from the Walter and Eliza Hall Institute (Melbourne, Australia). B6.*Sting*^–/–^ mice (33) were provided by Rachel Kuns (QIMR Berghofer Medical Research Institute). Transgenic PbTII mice (30, 31) were crossed to B6.*Ptprca* (CD45.1^+^) mice to generate PbTII × B6.*Cd45.1* (PbTII^WT^; CD45.1^+^CD45.2^+^). B6.*Sting*^–/–^ mice were crossed with PbTII mice to generate *Sting*-deficient PbTII mice (PbTII*^Sting^*; CD45.1^–^CD45.2^+^). PbTII mice were also crossed to B6.*Ifnar*^–/–^ mice to generate *Ifnar*-deficient PbTII mice (PbTII*^Ifnar^*; CD45.1^–^CD45.2^+^). All mice were housed under pathogen-free conditions at the QIMR Berghofer Medical Research Institute Animal Facility.

*Plasmodium berghei* ANKA infections were established from parasites passaged in C57BL/6J mice. Transgenic *P*. *berghei* ANKA (231c11) parasites (200 μl, in-house laboratory stock, frozen at –80°C) expressing luciferase and GFP (57) were thawed at room temperature (RT) and injected via the intraperitoneal route into a passage mouse. Three days p.i., 1 drop of blood was collected into 250 μl RPMI/PS with 1 IU/ml heparin from passage mice. Then, 50 μl of this blood suspension was stained with 10 μg/mL Hoechst 33342 (Sigma Aldrich) and 5 μM SYTO 84 (Sigma Aldrich) in RPMI/PS for 22 minutes at RT. Next, 300 μl of RPMI/PS was added, and each sample was acquired on a BD LSRFortessa (BD Biosciences). The pRBCs were identified as Hoechst 33342^+^SYTO 84^+^. The passage mouse was sacrificed at greater than 1% pRBCs on day 3 p.i. Blood was collected from the passage mouse by cardiac puncture into RPMI/PS containing 1 IU/ml heparin and centrifuged at 290*g* for 7 minutes at RT. RBCs were counted on a hemocytometer (Pacific Laboratory Products). A parasite inoculum containing 5 × 10^5^ pRBCs per ml was prepared, and mice were injected with 200 μl of the inoculum (1×10^5^ pRBCs) intravenously via the lateral tail vein. Blood parasitemia was measured as described above, while parasite biomass was calculated by measuring luciferase transgenic parasites in live mice, as previously performed (58).

### Preparation of splenic single-cell suspension.

Mouse spleens were collected and placed in 1% (v/v) FCS in PBS (1% FCS/PBS). Spleens were then mechanically processed through a 100 μm EASYstrainer Cell Strainer (Greiner Bio-One) using the back of a 5 ml syringe plunger (Terumo Medical). Cells were resuspended in 1% FCS/PBS and centrifuged at 350*g* before being lysed with 1 ml Red Blood Cell Lysing Buffer Hybrid-Max (Sigma Aldrich) for 5 minutes at RT. Cells were then washed with 10 ml of 1% FCS/PBS, resuspended in 5 ml 1% FCS/PBS, and stored at 4°C until required.

### Cotransfer of PbTII cells into recipient mice.

Splenic CD4^+^ T cells were isolated by MACS using the mouse CD4^+^ T Cell Isolation Kit (Miltenyi Biotec) according to the manufacturer’s instructions, and 100 μl of a single cell suspension was stained with 1 μg/ml CD4 BUV395, TCR-β BUV737, CD45.1 FITC, and CD45.2 BV711 (Supplemental Table 1) to check cell purity (>90%). Then PbTII*^Sting^* or PbTII*^Ifnar^* cells were mixed with PbTII^WT^ cells at a 1:1 ratio and diluted to 5 × 10^6^ cells/ml in RPMI/PS. A 200 μl cell suspension (containing 10^6^ cells) was injected intravenously into B6.*Ptprca* (CD45.1^+^) recipient mice.

### Flow cytometry.

Human cell/flow cytometry staining was performed in Falcon 96-Well Clear Round Bottom Tissue Culture-Treated Cell Culture Microplates (Corning Inc.). Cells were washed and incubated with LIVE/DEAD Fixable Blue (Life Technologies), monocyte blocker (Invitrogen), and Fc receptor (FcR) True Block (Invitrogen) for 12 minutes at 37°C. Cells were then washed and incubated with fluorescently conjugated surface-staining antibodies (Supplemental Table 1) for 30 minutes at 37°C. Cells were washed and fixed with BD Cytofix Fixation Buffer (BD Biosciences). After fixation, cells were washed twice and stained with fluorescently conjugated intracellular staining antibodies (Supplemental Table 1) for 40 minutes at RT. Cells were then washed twice and resuspended in 200 μl PBS at 4°C before being acquired on a 5-laser Cytek Aurora using SpectroFlo software, version 2.3 (Cytek Biosciences), then analyzed using FlowJo, version 10.7.1 (BD Biosciences).

### Prestain and stimulation of mouse Tr1 cells.

Mouse splenocytes were stained with 4 μg/ml LAG3 BV785 (BioLegend) and 4 μg/ml CD49b PeCy7 (BioLegend) in 30 μl PBS for 30 minutes at 37°C. After staining, 70 μl of complete media was added to the cell suspension (described above) along with 100 μl of complete media with 2× monensin, 25 ng/ml PMA, and 1.33 nM ionomycin and incubated for 3 hours at 37°C.

### Surface and intracellular staining.

Mouse prestained cells or spleen cells were washed once and incubated with TruStain FcR (BioLegend), True-Stain Monocyte Blocker (BioLegend), and LIVE/DEAD Fixable Aqua Dead Cell Stain (Life Technologies) for 15 minutes at 37°C. After incubation, cells were stained and fixed following the same procedures as used for the human sample processing described above.

### PCR for detection of genetic modifications.

DNA from CRISPR-edited and control cells was extracted by QuickExtract DNA Extraction Solution, according to the manufacturer’s instructions (Lucigen). The DNA was amplified with *TMEM173* primers (Supplemental Table 1), and 2 μl of genomic DNA (1:3 diluted), 5 μl 5× PCR buffer, 2 μl 25 mM MgCl_2_, 0.5 μl 10 mM dNTP, 0.5 μl 10 μM forward primer, 0.5 μl 10 μM reverse primer, and 0.125 U/μl GoTaq DNA Polymerase (Promega) were mixed to make a 25 μl PCR reaction mix (Supplemental Table 1). PCR was then performed in a T100 Thermal Cycler (Bio-Rad) using conditions outlined in Table 1.

### T7 endonuclease I mismatch assay.

After PCR amplification, 10 μl PCR products were incubated with 1.5 μl 10× NEBuffer (New England Biolabs) and 1.5 μl nuclease-free water with the thermal cycler setting at 10 minutes for 95°C, then 95°C–85°C (ramp rate of –2°C/second), and finally 85°C–25°C (ramp rate of –0.3°C/second). Then 13 μl PCR heteroduplexes were digested by 2 μl of 1 U/μL T7 Endonuclease I (New England Biolabs) at 37°C for 60 minutes. The digestion of CRISPR-edited DNA was visualized by running on a 5 % (w/v) agarose gel.

### Big dye sequencing.

PCR products (1–2 ng per 100 base pairs) were treated with ExoSAP-IT PCR Product Cleanup Reagent (Life Technologies) at a ratio of 5:2 at 37°C for 4 minutes, then 80°C for 1 minute. The PCR products were mixed with 6 pmol *TMEM173* forward primer (Supplemental Table 1) to make a final volume of up to 10 μl. Then the PCR products were submitted to the QIMR DNA-Seq facility for Big Dye sequencing.

### Western blotting.

Cells were lysed with 500 μl RIPA buffer (Cell Signaling Technology) with 1× PMSF (Cell Signaling Technology) and protease inhibitor cocktail (Cell Signaling Technology) per 10^7^ cells. According to the manufacturer’s protocol, the protein concentration was measured using a DC Protein Assay Kit II (Bio-Rad). The cell lysate was denatured at 75°C for 10 minutes, and the protein was separated by Bolt 4% to 12% (w/v) Bis-Tris Plus Gels (Life Technologies) and transferred onto a PVDF membrane (Millennium Science). The membrane was blocked with 50 ml Odyssey Blocking Buffer (LI-COR Bioscience) at RT for 1 hour and then incubated with 1/1,000 diluted anti-STING mAbs (Cell Signaling Technology) at 4°C overnight. On the second day, the membrane was washed and incubated with 0.2 μg/ml diluted IRDye 680RD donkey anti-mouse IgG secondary antibody and IRDye 800CW goat anti-rat IgG secondary antibody (LI-COR Bioscience, Supplemental Table 1) at RT for 30 minutes. The signal was detected using the Licor Odyssey CLx (Millennium Science).

### Cytometric bead array.

A human Th1/Th2/Th17 Cytokine Kit (BD Biosciences) was used to measure the production of cytokines per the manufacturer’s instructions. Assays were run on a 4 laser BD LSRFortessa Cell Analyzer (BD Biosciences). Cytometric bead array (CBA) data were analyzed using BD CBA FCAP Array software, version 3.0.

### RNA extraction.

Cells were washed and lysed with 350 μl RLT buffer (QIAGEN). RNA was extracted using the RNeasy Mini Kit (QIAGEN) according to the manufacturer’s instructions. The concentration of RNA (ng/μl) and sample purity (260/280 ratio) was measured using the NanoDrop 2000 UV-Vis Spectrophotometer (Thermo Fisher Scientific). Extracted RNA was reverse transcribed to complementary DNA (cDNA) using the High-Capacity cDNA Reverse Transcription Kit (Applied Biosystems) per the manufacturer’s instructions.

### RT-qPCR.

GoTaq qPCR Master Mix (Promega) was used in a final reaction volume of 10 μl containing 10 ng of template cDNA. Real-time quantitative PCR (RT-qPCR) was performed in Hard-Shell 384-Well Plates, thin wall, skirted, clear/clear (Bio-Rad), sealed with Microseal B PCR Plate Sealing Film (Bio-Rad) on the QuantStudio 5 Real-Time PCR System (Applied Biosystems). Relative quantification was performed using the comparative C_T_ method relative to the housekeeping gene *18S* rRNA because it was shown to be a stable housekeeping gene for human T lymphocytes (59).

### Ingenuity pathway analysis.

Gene ID, log_2_ fold-change (logFC) of gene expression, and adjusted *P* values were used to input into Ingenuity Pathway Analysis (IPA) (version 43605602; QIAGEN). Initial interrogation of each data set was performed using default values and parameters set on IPA. Upstream pathway analysis was performed on *IL10*. The tool *Grow* was used, direct and indirect interactions were selected, and the molecules/gene interactions related to the RNA-Seq data were identified.

### Statistics.

Statistical analysis was performed using GraphPad Prism 6 (GraphPad Software). Analysis of human cellular assays was performed using a 2-tailed, paired *t* test and 1-way or 2-way ANOVA with Šídák’s multiple-comparison test, as appropriate. Analysis of mouse flow cytometry data was performed using a 2-tailed paired *t* test. *P* values of less than 0.05 were considered significant.

### Study approval.

Human studies were undertaken at Q-Pharm Pty. Ltd. (Brisbane, Australia) under the approval of the QIMR Berghofer Human Research Ethics Committee (approval P1479). Written, informed consent was received from all participants. All studies were registered with the Australian and New Zealand Clinical Trial Registration scheme or with US ClinicalTrials.gov (NCT03542149) (Table 2). PBMCs were obtained from either healthy volunteers (QIMR Berghofer Medical Research Institute laboratory members) or volunteers participating in a CHMI study with *P*. *falciparum* (registration number NCT03542149; Table 2). Experimental mouse use was in accordance with the *Australian Code of Practice for the Care and Use of Animals for Scientific Purposes* (Australian National Health and Medical Research Council [NHMRC]) and was approved by the QIMR Berghofer Medical Research Institute Animal Ethics Committee; approval P2304).

### Data availability.

All data are available in the main text and supplemental materials, and values for all data points in graphs are reported in the Supporting Data Values file. Human RNA-Seq data have previously been published (21) and are available in the European Genome-Phenome Archive (EGA) database (https://ega-archive.org/) under accession number EGAS00001004454.

## Author contributions

YW and CRE conceptualized the study. YW, FDLR, CLE, TCMF, JAE, LB, JN, SSN, DC, MMDO, PTB, MSFS, DA, JRL, JZ, FHA, and CRE developed methodology and performed experiments and human studies. CRE, BEB, JSM, MJB, and JAL secured funding for the research. CRE, MJB, BEB, and JSM administered the project. FDLR, JAE, DA and FHA supervised the research. CRE and YW wrote the original draft manuscript, and CRE, YW, JAE, MJB, JAL reviewed and edited the paper.

## Supplementary Material

Supplemental data

Supporting data values

## Figures and Tables

**Table 2:**
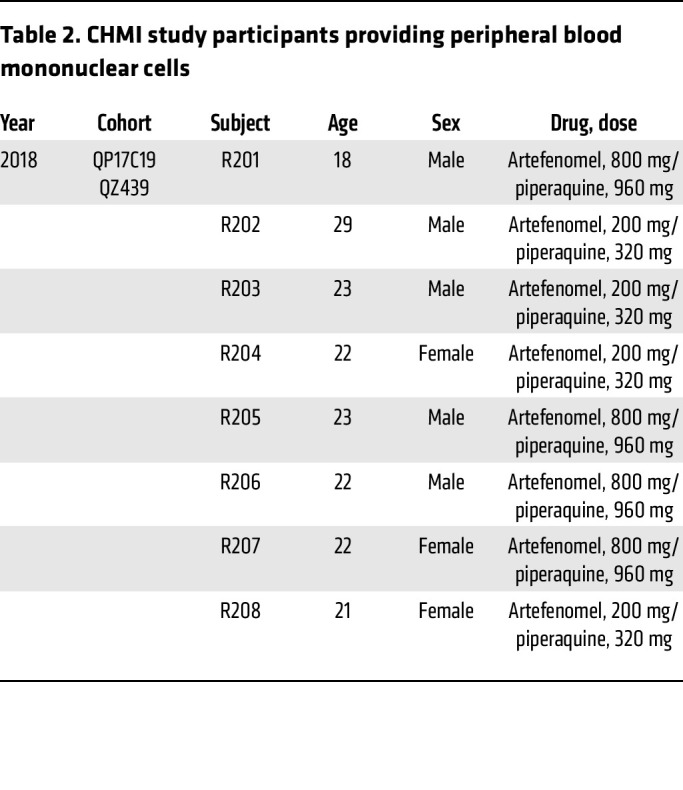
CHMI study participants providing peripheral blood mononuclear cells

**Table 1:**
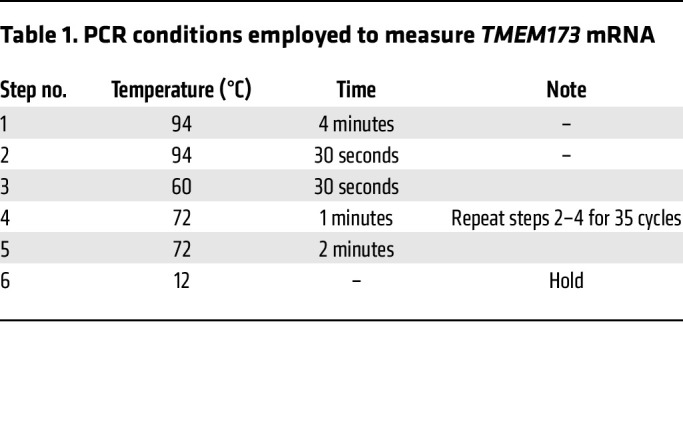
PCR conditions employed to measure *TMEM173* mRNA

## Version 1 10/02/2023

Electronic publication
