# Supplementary material for: STING activation promotes autologous type I interferon–dependent development of type 1 regulatory T cells during malaria
Source: J Clin Invest. 2023 Oct 2;133(19):e169417. doi: 10.1172/JCI169417 (PMC10541195; doi:10.1172/JCI169417)
Supplement: Supplemental data [file jci-133-169417-s198.pdf]

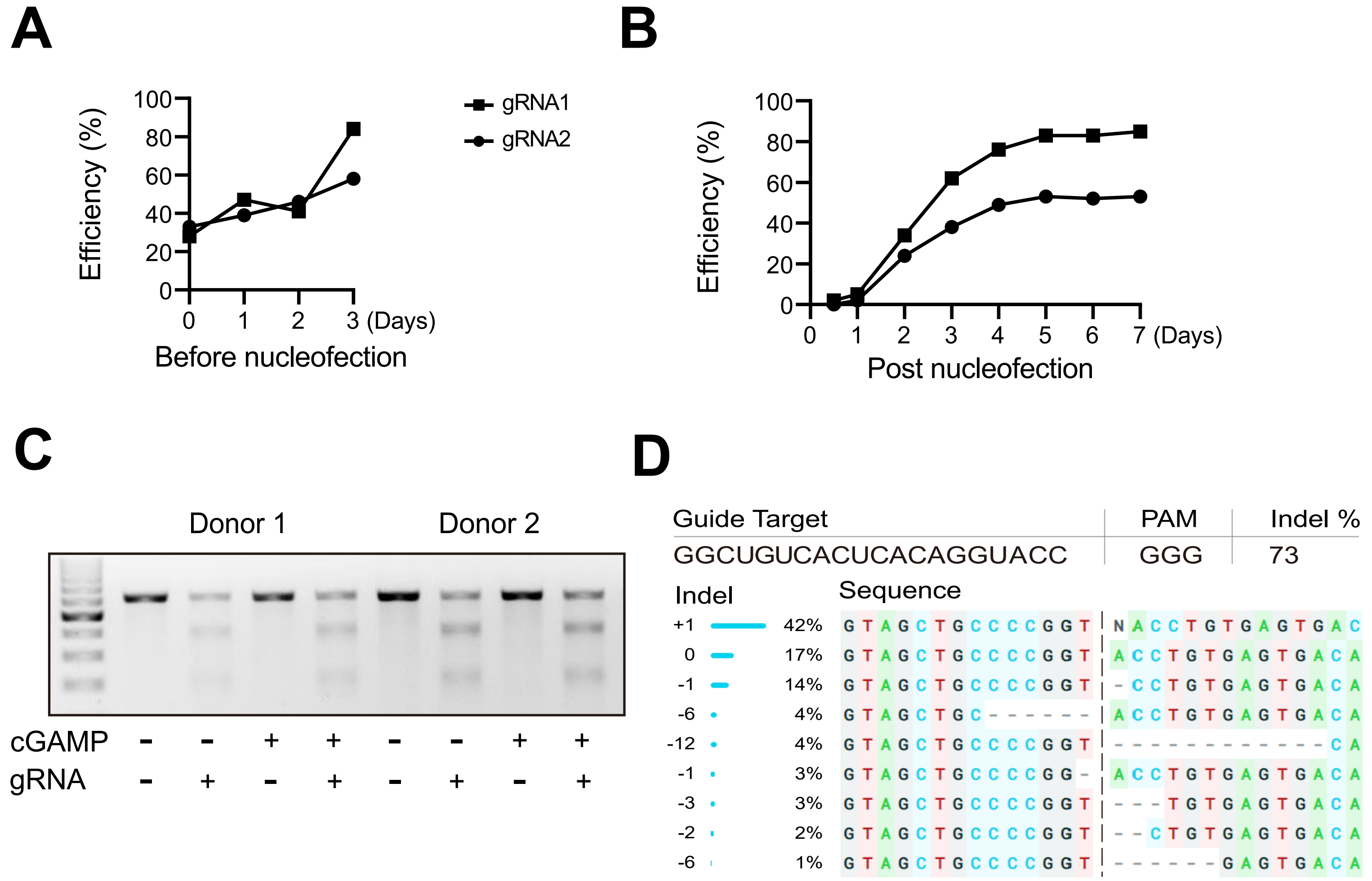

Figure S1. Optimization of the CRISPR/Cas9 gene editing protocol for human activated CD4⁺ T cells. **A**, Peripheral blood CD4⁺ T cells from a healthy volunteer were stimulated with αCD3ε and αCD28 mAbs plus IL-2 for 0, 1, 2 and 3 days before nucleofection with two different *TMEM173* guide RNAs (gRNA), and were stimulated for another 3 days, as above, before analysis. Gene-editing efficiency was measured by Big Dye Sequencing. **B**, CD4⁺ T cells from healthy volunteers were stimulated with αCD3ε and αCD28 mAbs plus IL-2 for 3 days before nucleofection with two different *TMEM173* gRNA and were then stimulated for 0 to 7 days before Big Dye Sequencing to calculate the efficiency of gene editing. **C**, CD4⁺ T cells were stimulated for 3 days, as above, transfected with *TEMEM 173* gRNA1, then stimulated for another 3 days with the addition of cGAMP 18 hours before analysis, as indicated. Representative T7 endonuclease I mismatch assay showing *TMEM173* modifications. **D**, Representative Inference of CRISPR Edits (ICE) analysis (https://ice.synthego.com/#/) of Big Dye Sequencing data showing the editing efficiency, deletions or insertion of single nucleotides or fragments (Indels).

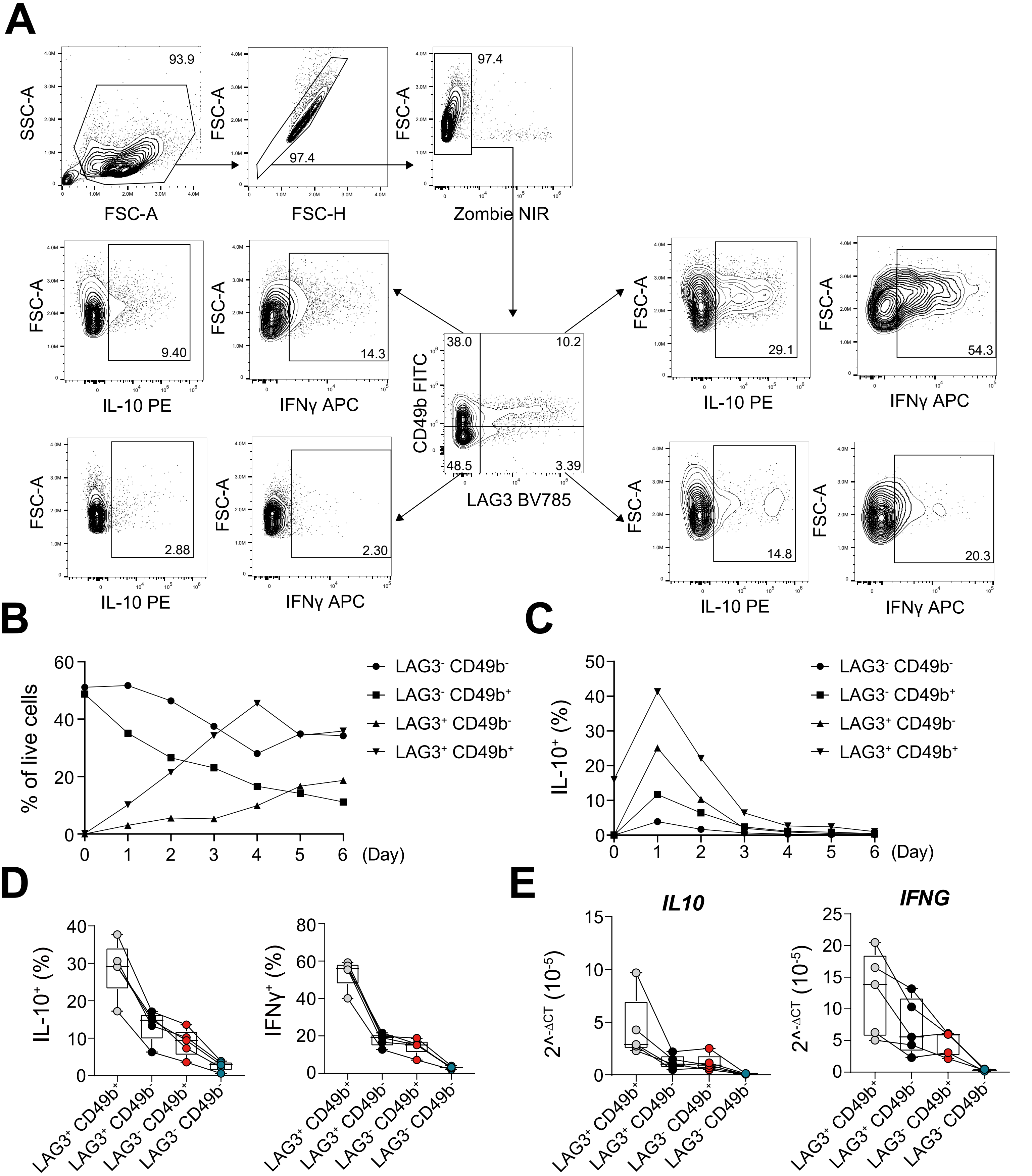

Figure S2. LAG3 and CD49b are surrogate markers for Tr1 cells. A, Peripheral blood CD4+ T cells from healthy volunteers (n = 4) were stimulated with αCD3ε and αCD28 mAbs plus IL-2 for 0 to 6 days. LAG3, CD49b, IL-10 and IFNγ expression was measured every day. The gating strategy for IL-10+, IFNγ+, and LAG3+ CD49b+ CD4+ T cells is shown. **B**, Changes in the frequency of LAG3+ CD49b+ CD4+ T cells from day 0 to day 6. **C**, The kinetics of IL-10 expression by CD4+ T cell subsets based on LAG3 and CD49b expression. **D**, Purified CD4+ T cells from healthy volunteers were stimulated with αCD3ε and αCD28 mAbs plus IL-2 for 1 day. Half of the cells were stained for LAG3, CD49b, IL-10 and IFNγ, while the other half were sorted into CD4+ T cell subsets based on LAG3 and CD49b expression. The expression of IL-10 and IFNγ protein by cell subsets is shown. **E**, *IL10* and *IFNG* mRNA in sorted LAG3 and CD49b CD4+ T cell subsets was measured by qPCR. Data was normalized to the housekeeping gene *18S* rRNA.

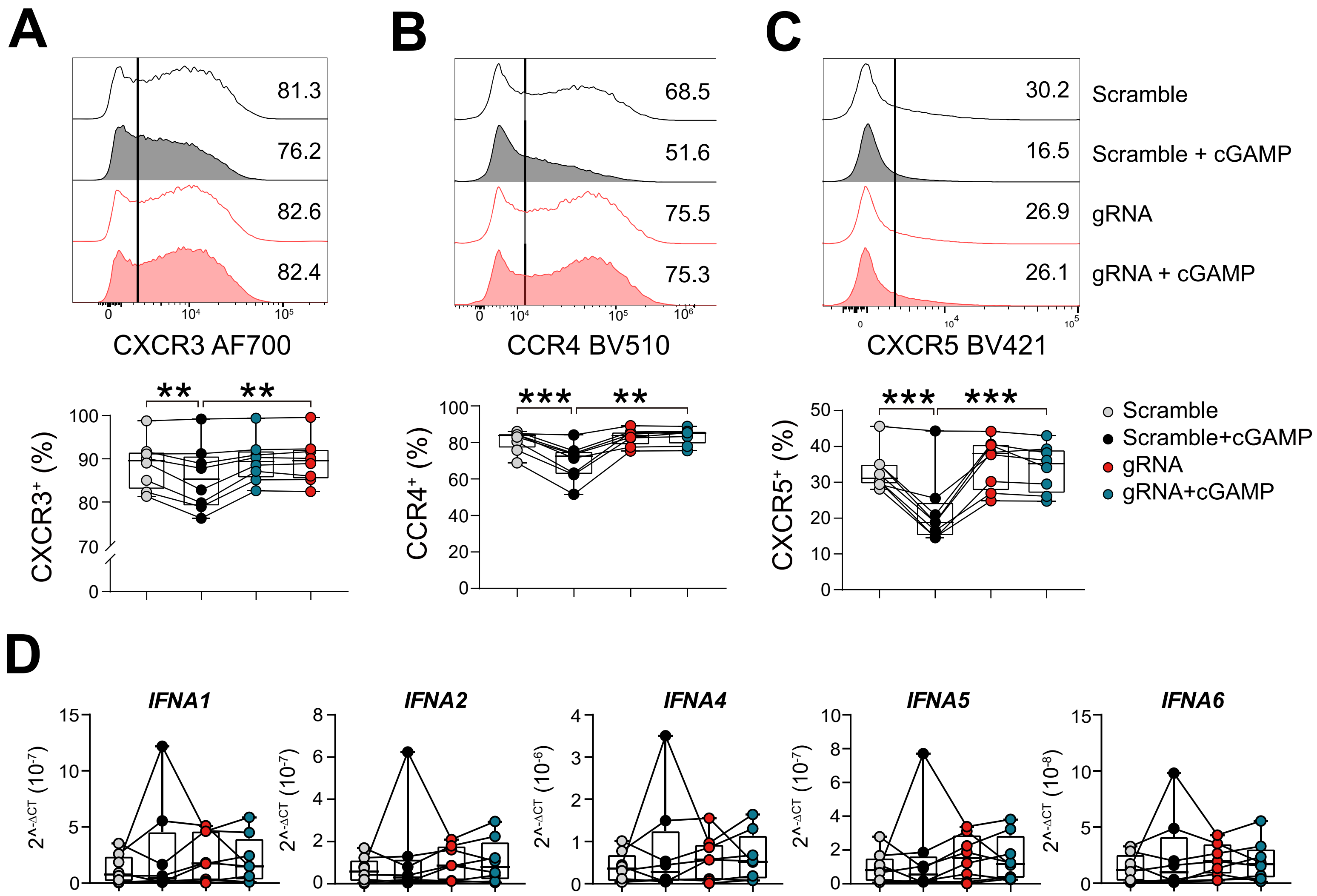

Figure S3. STING inhibits the expression of CXCR3⁺, CCR4⁺ and CXCR5⁺ by CD4⁺ T cells. A, B, C, Representative histograms and enumeration showing the frequency of $CXCR3^+$, $CCR4^+$ and $CXCR5^+$ $CD4^+$ T cells, respectively, following CRISPR/Cas9 to modify *TMEM173* expression. **D,** *IFNA1*, *IFNA2*, *IFNA4*, *IFNA5* and *IFNA6* mRNA was measured by qPCR. Data was normalized to the housekeeping gene *18S* rRNA. **A, B, C** and **D**: n=8, repeated measures two-way ANOVA with Šídák's multiple comparisons test, **P < 0.01 and ***P < 0.001.

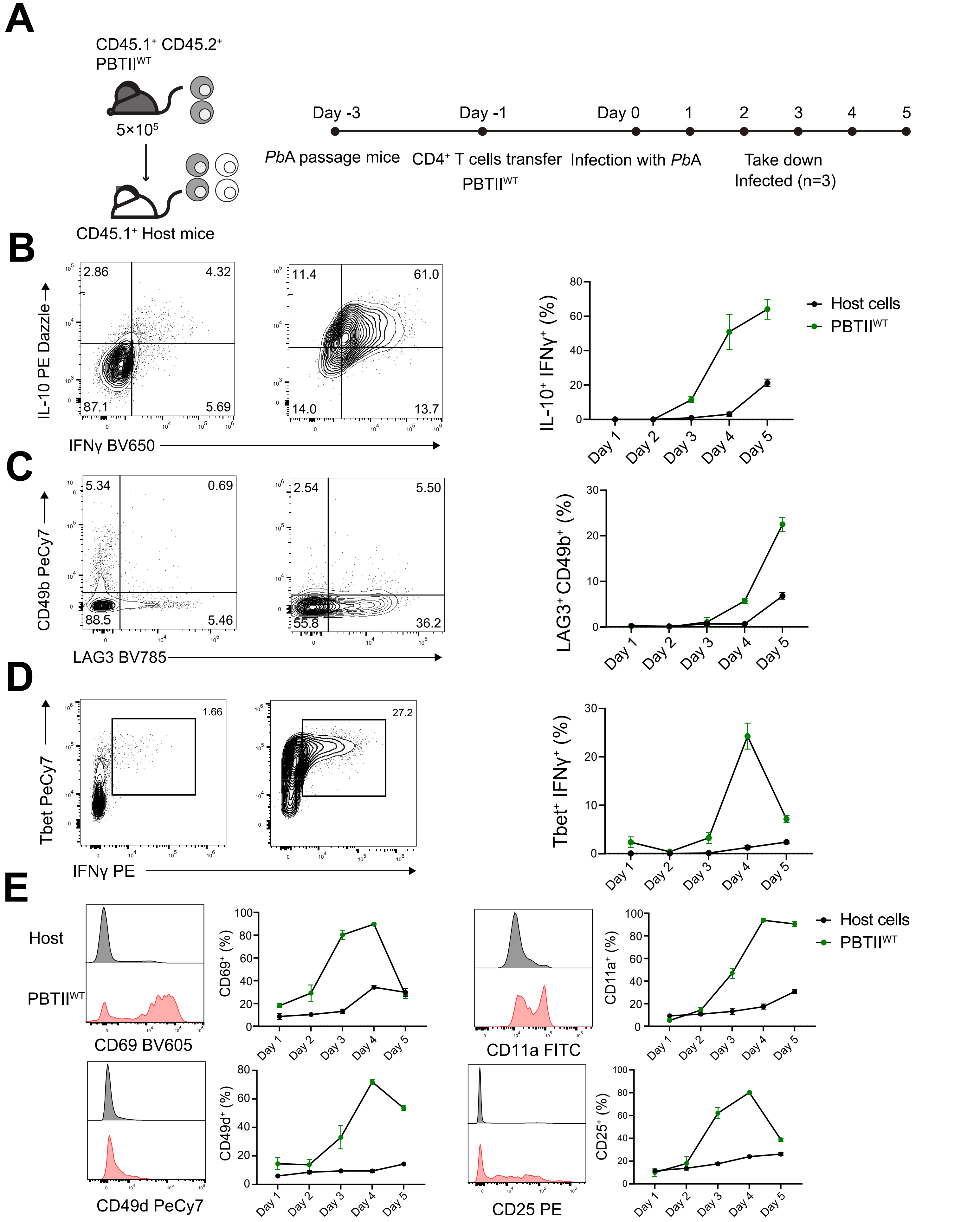

Figure S4. The kinetics of immune PbTII cell expansion in response to *Plasmodium berghei* ANKA (*Pb*A) infection. **A**, 5×10^5 CD45.1⁺ CD45.2⁺ PbTII^{WT} cells were transferred into the *ptprca* (CD45.1⁺) host mice at day -1. The mice were infected with *Pb*A on day 0 and were taken down from day 1 to day 5 post-infection (p.i.). **B, C, D,** Representative plots and enumeration showing the frequencies of IL-10⁺ IFNγ⁺, LAG3⁺ CD49b⁺ cells and Tbet⁺ IFNγ⁺ PbTII ^{WT} cells from day 1 to 5 p.i.. **E**, Representative histograms and enumeration showing the expression of the activation markers CD69, CD11a, CD49d and CD25 by PbTII^{WT} cells from day 1 to 5 p.i..

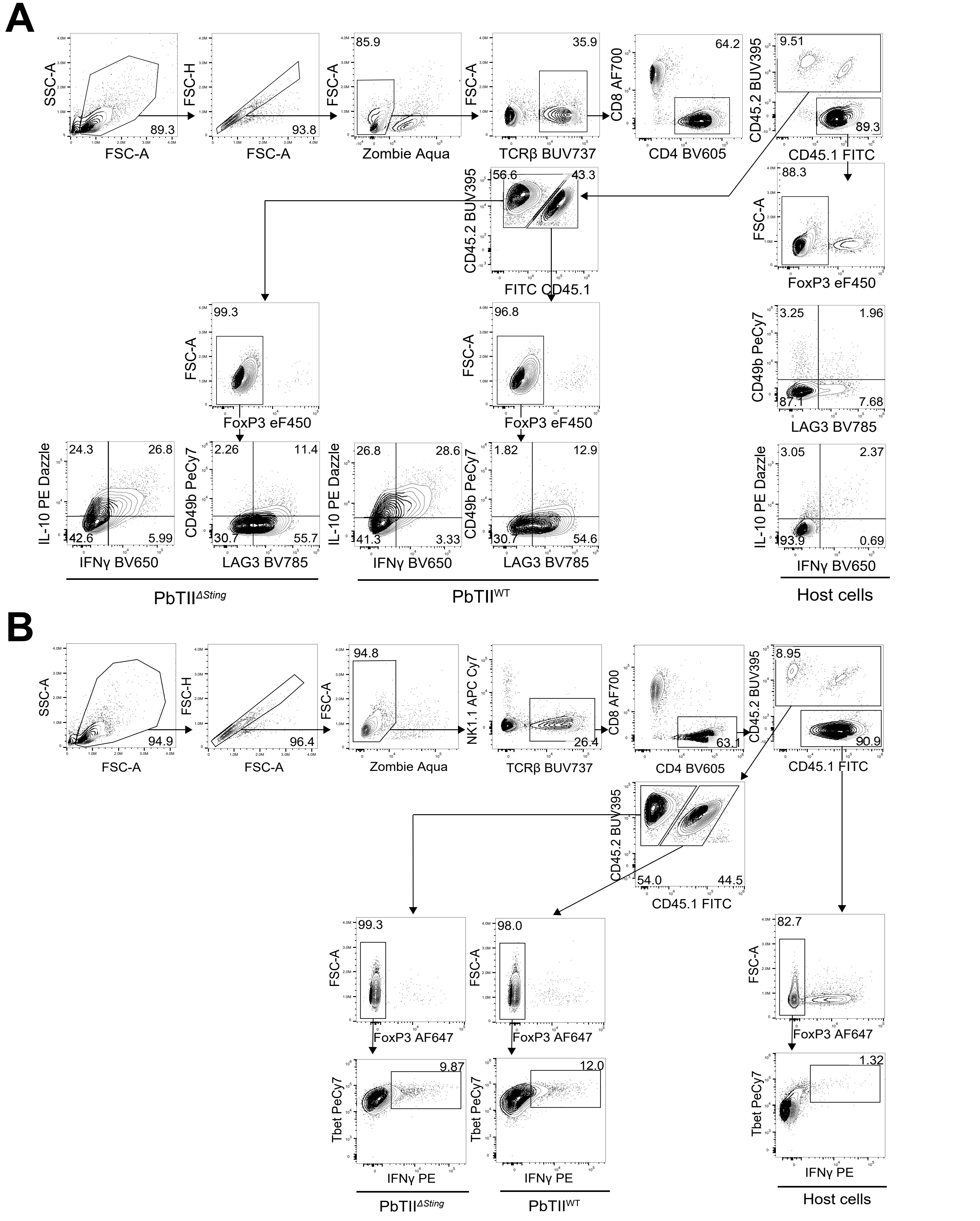

Figure S5. Identification of Tr1 and Th1 PbTII$^{\Delta Sting}$ and PbTII^{WT} cells. Live cells were selected, followed by gating on CD4^{+} T cells. These cells were then divided into adoptively transferred PbTII$^{\Delta Sting}$ and PbTII^{WT} cells, based on congenic marker expression. **A**, IL-10^{+} IFNγ^{+} and LAG3^{+} CD49d^{+} PbTII$^{\Delta Sting}$ and PbTII^{WT} Tr1 cells, and **B**, Tbet^{+} IFNγ$_{+}$ PbTII$^{\Delta Sting}$ and PbTII^{WT} Th1 cells were then assessed.

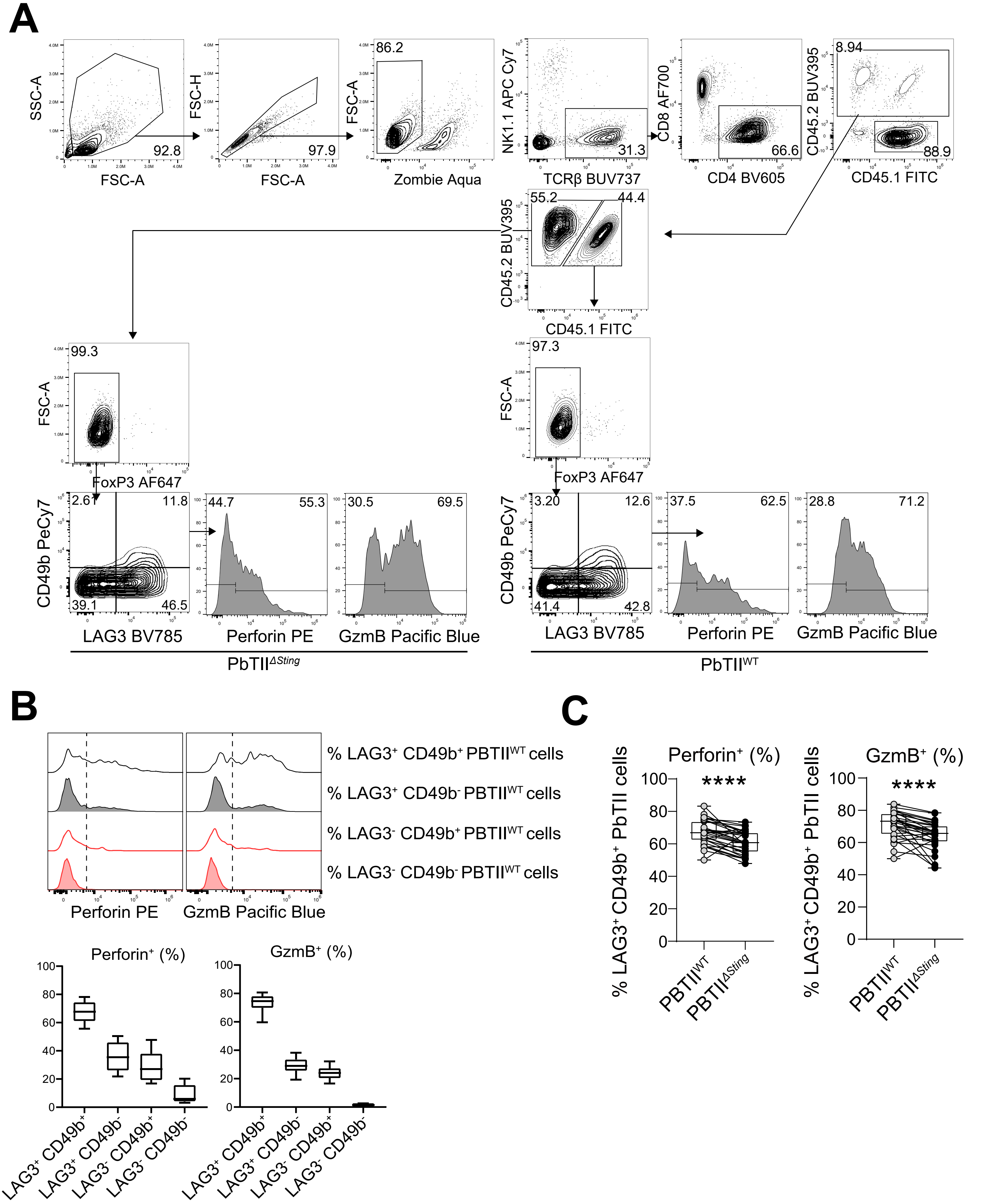

Figure S6. STING promotes the expression of cytotoxic molecules by Tr1 cells in experimental malaria. Spleen cells from day 4 post-infection with *Plasmodium berghei* ANKA were stimulated with monensin, PMA and ionomycin for 3 hours *ex vivo* before antibody staining. **A**, The gating strategy used to assess the frequency of Tr1 cells and their expression of cytotoxic molecules. **B**, Representative histograms and enumeration showing the expression of perforin and granzyme B (GzmB) by PbTII^{WT} cell subsets based on LAG3 and CD49b expression. **C**, The enumeration showing the frequency of perforin^{+} or GzmB^{+} PbTII$^{\Delta Sting}$ and PbTII^{WT} Tr1 cells. n = 15/group, pooled from 3 independent experiments, two-tailed paired t-test, P<0.001.

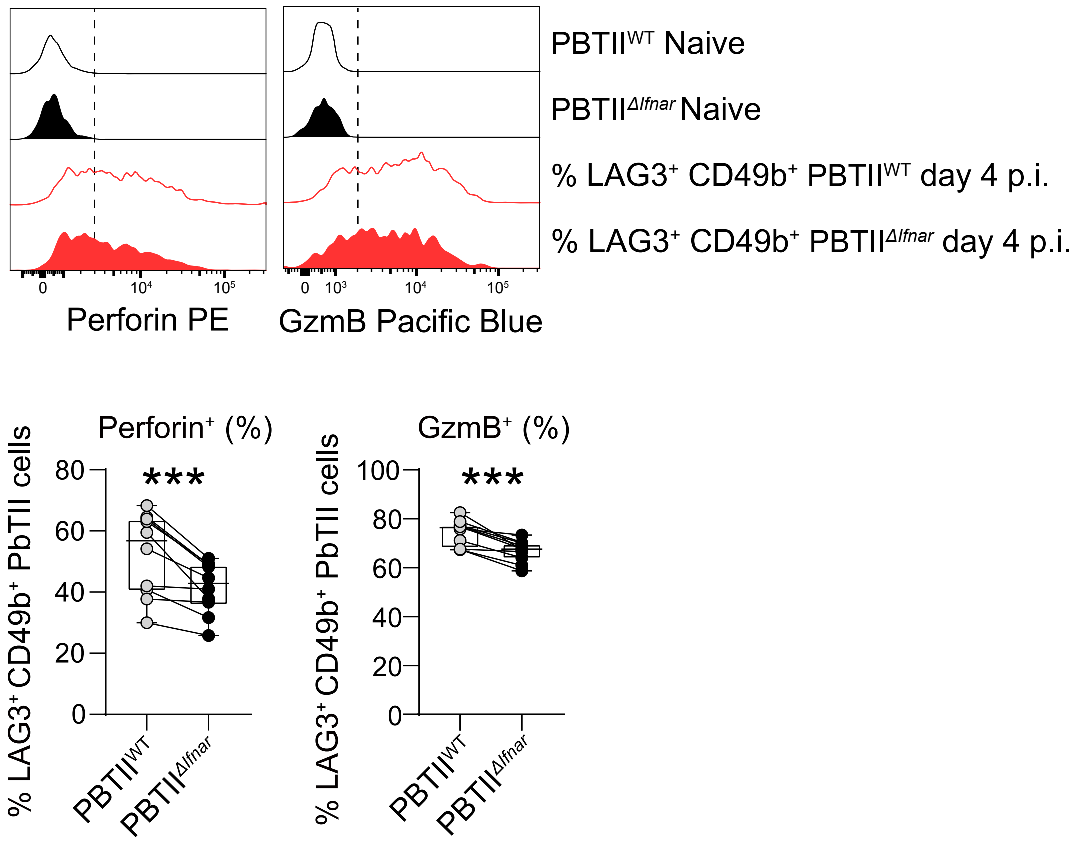

Figure S7. Type I IFN signalling promotes the expression of cytotoxic molecules by Tr1 PbTII cells during malaria. Spleen cells from day 4 post-infection with *Plasmodium berghei* ANKA were stimulated with monensin, PMA and ionomycin for 3 hours *ex vivo* before antibody staining. Representative histogram and enumeration showing the frequencies of perforin$^+$ or granzyme B (GzmB)$^+$ Tr1 cells in PbTII$^{\Delta Ifnar}$ and PbTII^{WT} cells. n = 10/group, pooled from 2 independent experiments, two-tailed paired t-test, P<0.001.

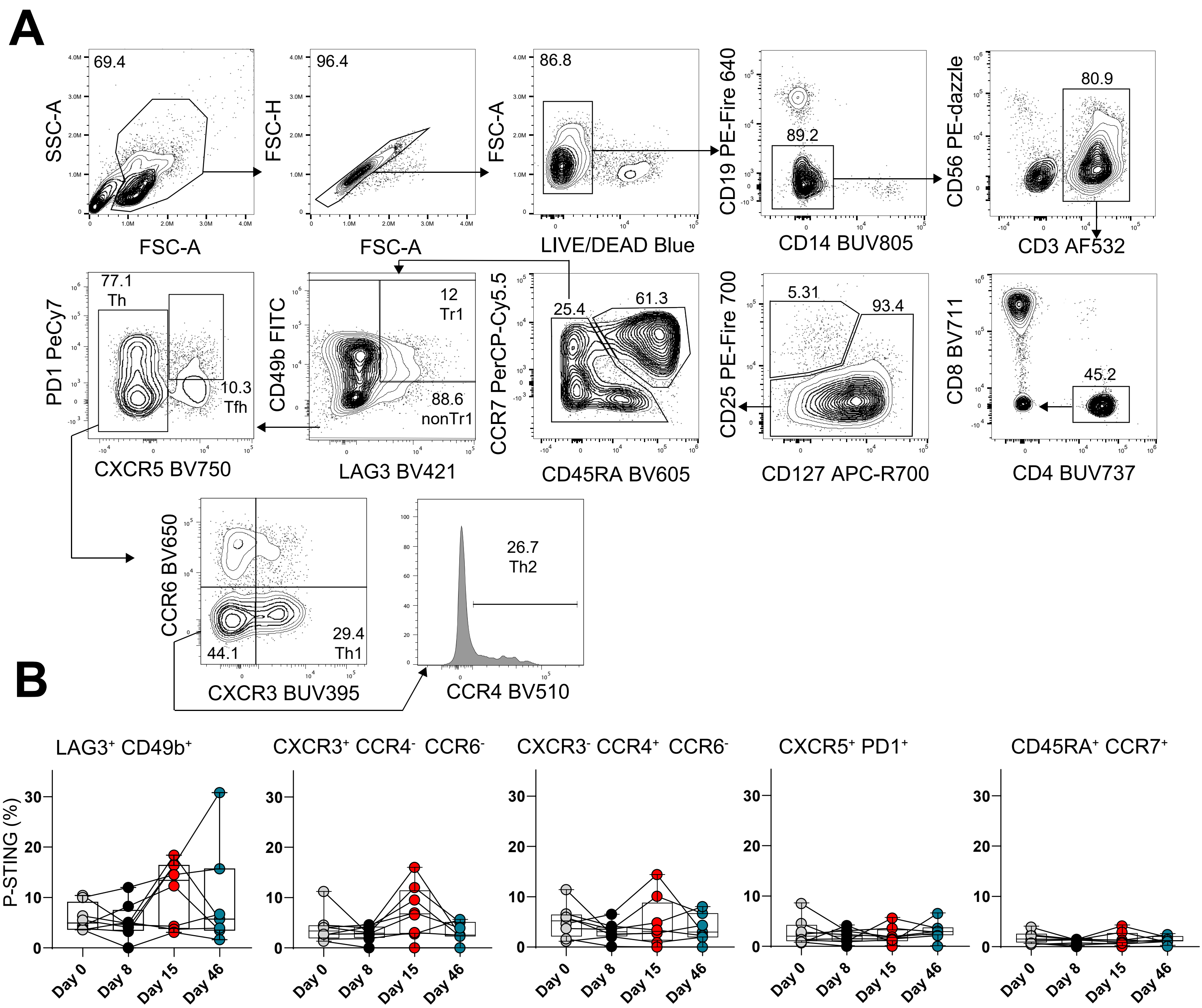

Figure S8. Tr1 cells from humans infected with *Plasmodium falciparum* are more sensitive to STING activation. A, The gating strategy used to identify $CD4^+$ T cell subsets. B, The frequency of different Th cell subsets that contained phosphorylated STING (P-STING) from day 0 post-infection (p.i.) to 46 p.i. in the presence of cGAMP for 18h.

Supplemental Table 1. Key resources.

REAGENT or RESOURCE	SOURCE	IDENTIFIER
Antibodies		
APC-R700 Mouse anti-human CD25	BD Horizon™	Cat#565106
Ultra-LEAF™ Purified anti-human CD3	BioLegend	Cat#317326
Ultra-LEAF™ Purified anti-human CD28	BioLegend	Cat#302934
Brilliant Violet 605™ anti-human CD366 (Tim-3) Antibody	BioLegend	Cat#345018
V450 Mouse Anti-Human CD127	BD Bioscience	Cat#560823
APC anti-human CD223 (LAG-3) Antibody	BioLegend	Cat#369212
Brilliant Violet 421™ anti-human CD223 (LAG-3) Antibody	BioLegend	Cat#369313
Brilliant Violet 785™ anti-human CD223 (LAG-3) Antibody	BioLegend	Cat#369322
Alexa Fluor® 647 Phospho-STING (Ser366) (D8K6H) Rabbit mAb	Cell Signaling Technology	Cat#43499
FITC anti-human CD49b Antibody	BioLegend	Cat#359305
Brilliant Violet 421™ anti-human CD279 (PD-1)	BioLegend	Cat#329920
PE/Dazzle™ 594 anti-human TIGIT	BioLegend	Cat#372716
beta Actin Loading Control Monoclonal Antibody (BA3R)	Life Technologies	Cat#MA5-15739
STING (D2P2F) Rabbit mAb	Cell Signaling Technology	Cat#13647

IRDye® 680RD Donkey anti-Mouse IgG Secondary Antibody	LI-COR Bioscience	Cat#926-68072
IRDye® 800CW Goat anti-Rat IgG Secondary Antibody	LI-COR Bioscience	Cat#926-32219
Alexa Fluor® 647 anti-mouse/rat/human FOXP3 Antibody	BioLegend	Cat#320014
Alexa Fluor® 700 anti-mouse CD8a Antibody	BioLegend	Cat#100730
APC/Cyanine7 anti-mouse NK-1.1 Antibody	BioLegend	Cat#108724
FITC anti-mouse CD45.1 Antibody	BioLegend	Cat#110706
PE anti-mouse IFN-γ Antibody	BioLegend	Cat#505808
PE/Cyanine7 anti-T-bet Antibody	BioLegend	Cat#644824
Brilliant Violet 650™ anti-mouse CD183 (CXCR3) Antibody	BioLegend	Cat#126531
Brilliant Violet 711™ anti-mouse CD45.2 Antibody	BioLegend	Cat#109847
APC anti-mouse TIGIT (Vstm3) Antibody	BioLegend	Cat#142106
APC/Fire™ 750 anti-mouse CD279 (PD-1) Antibody	BioLegend	Cat#135240
PerCP/Cyanine5.5 anti-mouse CD195 (CCR5) Antibody	BioLegend	Cat#107016
PE anti-mouse CD366 (Tim-3) Antibody	BioLegend	Cat#134004
PE/Dazzle™ 594 anti-mouse IL-10 Antibody	BioLegend	Cat#505034
PE/Cyanine7 anti-mouse CD49b Antibody	BioLegend	Cat#103518

Brilliant Violet 605™ anti-mouse CD152 Antibody	BioLegend	Cat#106323
Brilliant Violet 785™ anti-mouse CD223 (LAG-3) Antibody	BioLegend	Cat#125219
PE anti-mouse Perforin Antibody	BioLegend	Cat#154406
Pacific Blue™ anti-human/mouse Granzyme B Antibody	BioLegend	Cat#515408
FOXP3 Monoclonal Antibody (FJK-16s), eFluor 450	eBioscience™	Cat#48-5773-82
BV650 Rat Anti-Mouse IFN-γ	BD Horizon™	Cat#563854
BUV395 Rat Anti-Mouse CD4	BD Horizon™	Cat#563790
BUV737 Hamster Anti-Mouse TCR β Chain	BD Horizon™	Cat#612821
BUV395 Mouse Anti-Mouse CD45.2 (Clone 104 (RUO))	BD Horizon™	Cat#564616
Brilliant Violet 605™ anti-mouse CD4 Antibody (clone GK1.5)	BioLegend	Cat#100451
Zombie NIR™ Fixable Viability Kit	BioLegend	Cat# 423105
Anti-Interferon-α/β Receptor Chain 2 Antibody, clone MMHAR-2	Sigma-Aldrich	Cat#MAB1155
Mouse IgG2A Isotype Control, clone 20102	R&D Systems	Cat#MAB003
Recombinant Human IFN-β	PeproTech	Cat#300-02BC
Biological Samples		

PBMCs from healthy volunteers	QIMR Berghofer Medical Research Institute	N/A
Chemicals, Peptides, and Recombinant Proteins		
β-Mercaptoethanol	Sigma-Aldrich	Cat#M6250
Glutamax I, 100X	Life Technologies	Cat#35050061
BD Cytofix™ Fixation Buffer Set	BD Biosciences	Cat#554714
RPMI 1640 Media	Life Technologies	Cat#11875-093
Non-Essential Amino Acids Solution (100X)	Sigma-Aldrich	Cat#M7145
D-(+)-Glucose solution	Sigma-Aldrich	Cat#G8769
Sodium pyruvate solution	Sigma-Aldrich	Cat# S8636
ImmunoCult™ Human CD3/CD28/CD2 T Cell Activator	Stemcell Technologies	Cat#10970
RIPA Buffer (10X)	Cell Signaling Technology	Cat#9806S
PMSF	Cell Signaling Technology	Cat#8553
Protease Inhibitor Cocktail (100X)	Cell Signaling Technology	Cat#5871
eBioscience™ Foxp3/Transcription Factor Staining Buffer Set	Thermo Fisher Scientific	Cat#00-5523-00
Ficoll-Paque™ PLUS density gradient media	GE Healthcare	Cat#17144003

Bolt™ 4-12% Bis-Tris Plus Gels	Life Technologies	Cat#NW04125BOX
20X Bolt™ MES SDS Running Buffer	Life Technologies	Cat#B0002
Bolt™ Transfer Buffer (20X)	Life Technologies	Cat#BT0006
Bolt™ Antioxidant	Life Technologies	Cat#BT0005
DTT (Dithiothreitol)	Cell Signaling Technology	Cat#7016
Blue Loading Buffer Pack	Cell Signaling Technology	Cat# 7722
Immobilon-FL 10x 10cm Sheet 10pk PVDF 0.45um	Millennium Science	Cat#MMI-IPFL10100
Odyssey® Blocking Buffer in PBS	LI-COR Bioscience	Cat#927-40000
Human IL-2 IS, research grade	Miltenyi Biotec	Cat#130-097-742
GoTaq® qPCR Master Mix	Promega	Cat#A6001
ExoSAP-IT™ PCR Product Cleanup Reagent 100X	Life Technologies	Cat#78200
HEPES 1M	Life Technologies	Cat#15630080
iBright™ Prestained Protein Ladder	Life Technologies	Cat# LC5615
QuickExtract™ DNA Extraction Solution	Lucigen	Cat#QE0905T
Dimethyl sulfoxide Hybri-Max™	Sigma-Aldrich	Cat#D2650
GoTaq® Flexi DNA Polymerase	Promega	Cat# M8295
Trypan blue stain (0.4%) for use with Countess™ Automated Cell Counter	Invitrogen	Cat# T10282
Zombie Aqua™ fixable viability dye kit	BioLegend	Cat#423102

2’3’-cGAMP	Jomar Life Research	Cat#tlrl-nacga23-1
GeneRuler 100 bp DNA Ladder	Life Technologies	Cat#SM0241
Tween 20	Sigma-Aldrich	Cat#P1379
Fetal Bovine Serum, certified, United States	Life Technologies	Cat#16000044
N,N-Dimethylacetamide	Sigma-Aldrich	Cat#271012
INCB018424 (Ruxolitinib)	Chemietek	CT-INCB
Riamet 20/120 (20mg artemether and 120mg lumefantrine; AL)	Novartis	• N/A
Critical Commercial Assays		
P3 Primary Cell 4D-NucleofectorTM X Kit S	Lonza	Cat#V4XP-3032
DC™ Protein Assay Kit II	Bio-Rad	Cat#5000112
EasySep™ Human CD4+ T Cell Isolation Kit	Stemcell Technologies	Cat#17952
High-capacity cDNA Reverse Transcription Kit	Applied Biosystems	Cat#4368814
QIAshredder	QIAGEN	Cat#79654
RNase-free DNase Set	QIAGEN	Cat#79254
RNeasy Mini Kit	QIAGEN	Cat#74104
ISOLATE II Genomic DNA Kit	Bioline	Cat#BIO-52065
T7 Endonuclease	New England Biolabs	Cat#M0302S
NEBuffer™ 2	New England Biolabs	Cat#B7002S

IFNγ Secretion Assay – Detection Kit (APC), human	Miltenyi Biotec	Cat# 130-090-762
IL-10 Secretion Assay – Detection Kit (PE), human	Miltenyi Biotec	Cat# 130-090-434
Oligonucleotides		
Modified gRNA targeting the *TMEM173* locus (GGCUGUCACUCACAGGUACC)	Synthego	N/A
Modified gRNA targeting the *TMEM173* locus (ACAGGUACCGGGGCAGCUAC)	Synthego	N/A
Modified negative control gRNA	Synthego	N/A
RT^2 qPCR Primer Assay for Human *18SrRNA* 330001	QIAGEN	PPH05666E-200
RT^2 qPCR Primer Assay for Human *TMEM173*	QIAGEN	PPH18521A-200
RT^2 qPCR Primer Assay for Mouse *Tmem173*	QIAGEN	PPM29247A-200
RT^2 qPCR Primer Assay for Human *IL10*	QIAGEN	PPH00572C
RT^2 qPCR Primer Assay for Human *IFNG*	QIAGEN	Cat# PPH00380C
Human *TMEM173* Forward PCR primer (ACTTGGCCAGAGCTTCTACC)	Sigma-Aldrich	N/A
Human *TMEM173* Reverse PCR primer (GTCATGGATTTCTTGGTGCCC)	Sigma-Aldrich	N/A

Mouse *Ifnar ko* Forward PCR primer<br>(CAC TGG AGA AAC CTT CCT TC)	Sigma-Aldrich	N/A
Mouse *Ifnar ko* Reverse PCR primer<br>(CAG TAA GTA GTC TCT CTG GCA AG)	Sigma-Aldrich	N/A
Mouse *PbTII TCR α* Forward PCR primer<br>(GGATCCAGGAATGGACAAGATTCTG)	Sigma-Aldrich	N/A
Mouse *PbTII TCR α* Reverse PCR primer<br>(CAGATCTCAACTGGACCACAG)	Sigma-Aldrich	N/A
Mouse *PbTII TCR β* Forward PCR primer<br>(GGATCGATCACACTTGTTTTCCGTG)	Sigma-Aldrich	N/A
Mouse *PbTII TCR β* Reverse PCR primer<br>(GATCGATCAGCTCACCTAACACGAGGA)	Sigma-Aldrich	N/A
Mouse *Tmem173 neo/neo* Forward PCR primer<br>(CTG TGC TCG ACG TTG TCA CT)	Sigma-Aldrich	N/A
Mouse *Tmem173 neo/neo* Reverse PCR primer<br>(GCT CTT CGT CCA GAT CAT CC)	Sigma-Aldrich	N/A
Mouse *Tmem173 flpase* Forward PCR primer<br>(GCT GGG AAT TGA ACG TAG GA)	Sigma-Aldrich	N/A
Mouse *Tmem173 flpase* Reverse PCR primer<br>(GAG ACA AAG GCA AGC AC)	Sigma-Aldrich	N/A
Data sets		

Human RNAseq data set	European Genome-Phenome Archive (EGA) database (https:// ega-archive.org/)	accession # EGAS00001004454
Software and Algorithms		
FlowJo, v10 OSX	FlowJo, LLC. https://www.flowjo.com/	
EdgeR	Robinson, M.D., D.J. McCarthy, and G.K. Smyth, *edgeR: a Bioconductor package for differential expression analysis of digital gene expression data.* Bioinformatics, 2010. **26**(1): p. 139-40. https://bioconductor.org/packages/release/bioc/html/edgeR.html	

GraphPad Prism 7, v7.0c	GraphPad Software https://www.graphpad.com/scientific-software/prism/	
Ingenuity Pathway Analysis (IPA), version 43605602	QIAGEN https://www.qiagenbioinformatics.com/products/ingenuity-pathway-analysis/	
R Project for Statistical Computing	https://www.r-project.org/	RRID: SCR_001905
RStudio Desktop, v1.0.136 - 1.2.1335	https://www.rstudio.com/products/RStudio/	
Other		
tSNEplots, v1.4	*Ashhurst, T. M. (2017). tSNEplots v1.4. GitHub repository. DOI: TBC, repository: https://github.com/sydneycytometry/tSNEplots/releases.*	
